# An Air‐Stable, Neutral Phenothiazinyl Radical with Substantial Radical Stabilization Energy

**DOI:** 10.1002/chem.201905238

**Published:** 2020-02-19

**Authors:** Lukas M. Sigmund, Fabian Ebner, Christoph Jöst, Jonas Spengler, Nils Gönnheimer, Deborah Hartmann, Lutz Greb

**Affiliations:** ^1^ Anorganisch-Chemisches Institut Ruprecht-Karls-Universität Heidelberg Im Neuenheimer Feld 270 69120 Heidelberg Germany

**Keywords:** aminyl radicals, phenothiazines, radical stabilization energy, radicals, redox chemistry

## Abstract

The vital effect of radical states on the pharmacological activity of phenothiazine‐based drugs has long been speculated. Whereas cationic radicals of N‐substituted phenothiazines show high stability, the respective neutral radicals of N‐unsubstituted phenothiazines have never been isolated. Herein, the 1,9‐diamino‐3,7‐di‐*tert*‐butyl‐*N* ^1^,*N* ^9^‐bis(2,6‐diisopropylphenyl)‐10*H*‐phenothiazin‐10‐yl radical (SQH_2_^.^) is described as the first air‐stable, neutral phenothiazinyl free radical. The crystalline dark‐blue species is characterized by means of EPR and UV/Vis/near‐IR spectroscopy, as well as cyclic voltammetry, spectro‐electrochemical analysis, single‐crystal XRD, and computational studies. The SQH_2_^.^ radical stands out from other aminyl radicals by an impressive radical stabilization energy and its parent amine has one of the weakest N−H bond dissociation energies ever determined. In addition to serving as open‐shell reference in medicinal chemistry, its tridentate binding pocket or hydrogen‐bond‐donor ability might enable manifold uses as a redox‐active ligand or proton‐coupled electron‐transfer reagent.

## Graphical abstract

**Out in the open**: An air‐stable phenothiazinyl radical was synthesized and characterized by means of experiments, spectroscopy, and computational investigations. The crystalline dark‐blue compound shows addressable redox activity and represents the first N‐unsubstituted phenothiazine‐based aminyl radical.

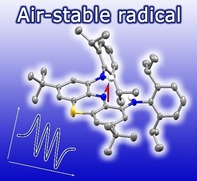

## Introduction

**Figure 1:**
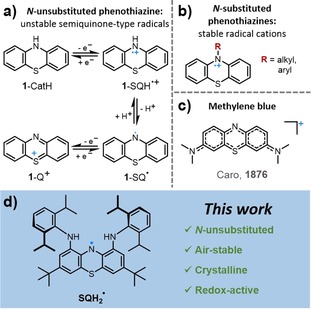
a) Redox chemical processes of N‐unsubstituted phenothiazine **1**, involving the catechol‐type oxidation state (CatH) that can undergo single‐electron oxidization to the semiquinonic radical cation (SQH^.+^). Deprotonation gives the neutral semiquinonic radical (SQ^.^), which easily oxidizes to the quinonic cation (Q^+^). b) Well‐known stable N‐substituted phenothiazinyl radical cations. c) Structure of the methylene blue cation. d) The 1,9‐diamino‐3,7‐di‐*tert*‐butyl‐*N* ^1^,*N* ^9^‐bis(2,6‐diisopropylphenyl)‐10*H*‐phenothiazin‐10‐yl radical (SQH_2_^.^), which is a new stable free aminyl radical described herein.

The phenothiazine core structure appears in many pharmacologically active molecules with, for example, anticancerogenic, antipsychotic, or cardiovascular effects.^1^ It has been suggested that radical states are responsible for the high activity of these drugs.^2^ Moreover, phenothiazine‐based aminyl radicals were observed during the selective cross‐dehydrogenative amination of phenols or anilines.^3^ Whereas the cationic radicals of N‐substituted phenothiazines^4^ are known for their high stability (Figure 1 b),^5^ a substantial drop in stability occurs without the substituent at the nitrogen atom. The N‐unsubstituted phenothiazines easily undergo stepwise two‐electron oxidation and deprotonation (Figure 1 a), wherein the semiquinonic radical (SQH^.^) state is vulnerable for disproportionation or oxidation into the favorable aromatic 14 π‐electron quinonic cation (Q^+^) state. Phenothiazine also constitutes the core structure of methylene blue, which is a well‐known dye first prepared in 1876 by Caro at BASF (Figure 1 c).^6^ Methylene blue was the first synthetic compound ever applied as an antiseptic agent in clinical therapy, even before the advent of sulfonamides or penicillin.^7^ The methylene blue radical was postulated in 1939 and later supported by evidence from combined electrochemical and EPR studies,^8^ which revealed a high tendency toward disproportionation.^9^ Although some persistent N‐unsubstituted phenothiazinyl radicals permitted spectroscopic characterization, the isolation of a crystalline and air‐stable derivative has, to the best of our knowledge, never been accomplished.3b, 10

Herein, we describe the air‐stable, neutral, N‐unsubstituted phenothiazinyl radical SQH_2_^.^ (Figure 1 d). The crystalline compound was characterized by means of single‐crystal X‐ray diffraction (SCXRD), EPR spectroscopy, elemental analysis, UV/Vis/near‐infrared (NIR) absorption spectroscopy, cyclic voltammetry, and spectro‐electrochemical analysis. The experimental data is compared and rationalized by DFT and time‐dependent (TD) DFT computations. SQH_2_^.^ is obtained as the sole product from the neutral precursor amine CatH_3_ with O_2_ (air) as the oxidant. Strikingly, CatH_3_ possesses one of the weakest hitherto determined N−H bond‐dissociation energies. In addition to ultimately serving as a radical reference within the field of phenothiazine‐based drugs, dyes, and reagents, SQH_2_^.^ expands the, to date, limited class of non‐heteroatom‐substituted aminyl free radicals that can be handled without any precautions.^11^

## Results and Discussion

**Figure 2:**
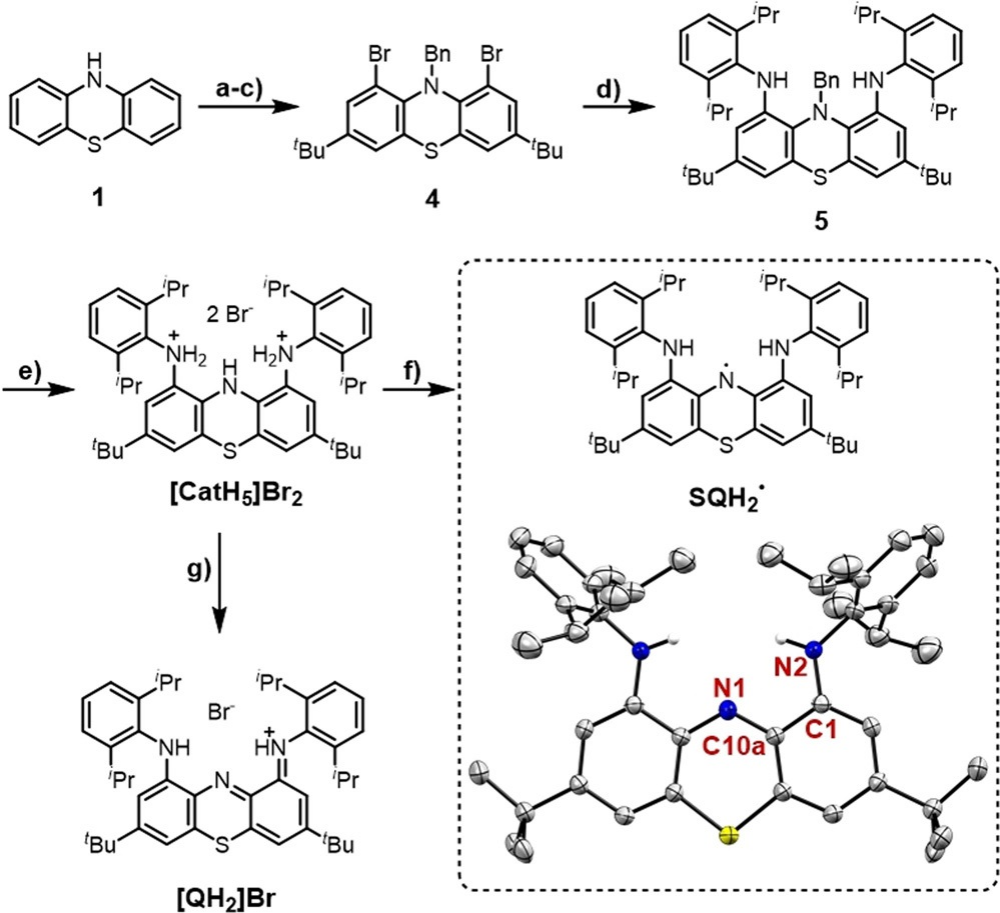
Synthesis and molecular structure of SQH_2_^.^ (displacement ellipsoids are shown at the 50 % probability level). Selected bond lengths [pm]: N2−C1 137.7(2), C1−C10a 142.7(3), C10a−N1 135.9(2). a) *t*BuCl, AlCl_3_, CH_2_Cl_2_, 0 °C, 15 min, 58 % yield; b) Br_2_, chloroform, 0 °C to RT, 3 h, 88 % yield; c) NaH, benzyl bromide (BnBr), THF, 60 °C, 17 h, 79 % yield; d) 10 mol % [Pd(dba)_2_] (dba=dibenzylideneacetone), 25 mol % P(*t*Bu)_3_, NaO*t*Bu, toluene, reflux, 2.5 h, 43 % yield; e) HBr_aq_, EtOAc, 60 °C, 1 h, 41 % yield; f) Cs_2_CO_3_, H_2_O, air, CH_2_Cl_2_, RT, 1.5 h, quant.; g) H_2_O, air, CH_2_Cl_2_, RT.

SQH_2_^.^ was prepared within six linear steps, starting from unsubstituted 10*H*‐phenothiazine (**1**), and without the need for column chromatography (Figure 2 a). N‐Benzyl‐protected **4** was obtained by procedures adapted from the literature.^12^ The diisopropylphenyl groups were introduced through Buchwald–Hartwig amination with a bis(dba)palladium(0)/tris‐*tert*‐butylphosphine catalytic system. A defined reaction time of 2.5 h at 110 °C in toluene was crucial to achieve a maximum yield of 43 % for this challenging double cross‐coupling reaction. The benzyl protecting group was removed with concentrated hydrobromic acid to yield the dicationic ammonium bromide, [CatH_5_][Br]_2_ (for SCXRD results, see the Supporting Information). The final target was obtained by dissolving [CatH_5_][Br]_2_ and cesium carbonate in dichloromethane/water (100:1) together with a flow of air through the reaction mixture.

Deprotonation and one‐electron oxidation immediately caused a color change from pale orange to intense blue. SQH_2_^.^ was isolated in quantitative yield as dark blue crystals, which did not show any sign of decomposition under ambient conditions or in basic/neutral solution. Purity of the compound was verified by means of elemental analysis. The effective magnetic moment of SQH_2_^.^ was determined by using the Evans NMR spectroscopy method,^13^ yielding 1.62 *μ*_B_, which was close to the expected spin‐only value for *S*=1/2 (1.73 *μ*_B_).^13^ Single crystals of SQH_2_^.^ suitable for XRD analysis were grown from a saturated solution in ethanol (Figure 2). The obtained structural parameters were compared with the DFT‐calculated (TPSS‐D3(BJ)/def2‐TZVPP) geometric data of the three possible redox states (Cat/SQ^.^/Q) and were found to match ideally with those for SQ^.^ (see the Supporting Information for a detailed comparison). The two‐electron‐oxidized species [QH_2_]Br was formed by air oxidation of [CatH_5_][Br]_2_ under nonbasic conditions or with NOSbF_6_ as an oxidant and was characterized crystallographically (Figure 2; see the Supporting Information for SCXRD results). Herein, the characteristic C1−N2/C10a−N1 bonds are shortened (133.4/132.5 pm) in comparison with those of SQH_2_^.^. Again, the structural parameters are in perfect agreement with the calculated geometry of [QH_2_]Br, giving further support for the correct assignment of the respective oxidation states.

**Figure 3:**
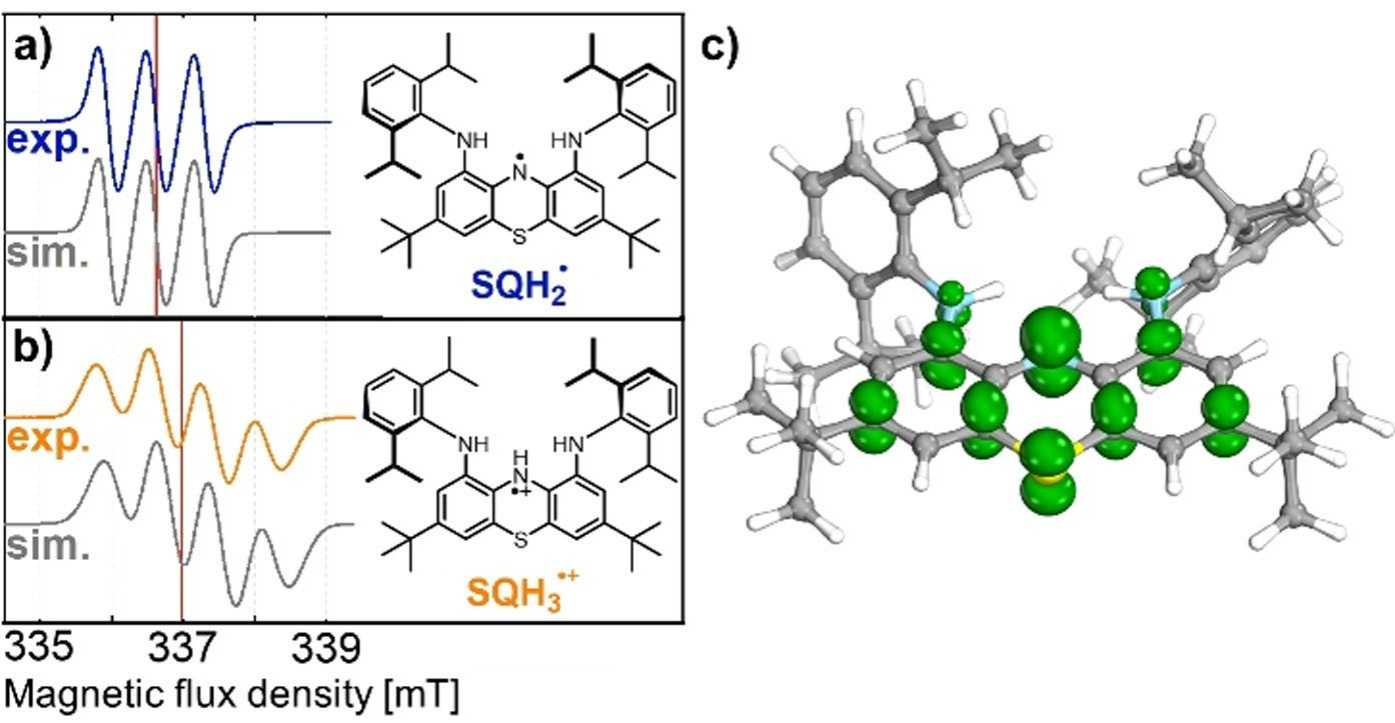
Measured and simulated solution X‐band EPR spectra of a) SQH_2_^.^ and b) SQH_3_^.+^. The red lines mark the center of the signals at 336.624 and 336.994 mT, respectively. c) Computed α spin‐density distribution of SQH_2_^.^ (isosurface threshold: 0.004).

**Figure 4:**
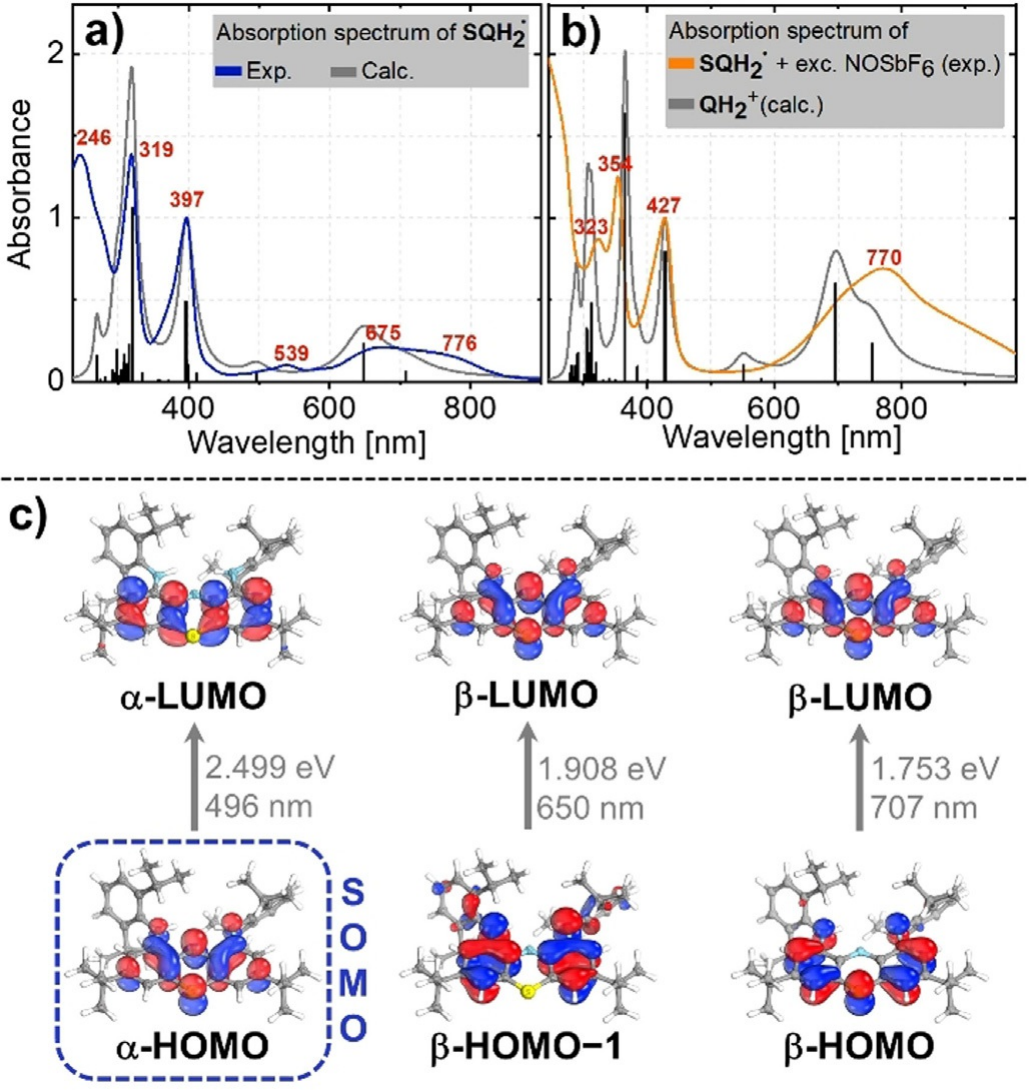
a) UV/Vis/NIR spectrum of SQH_2_^.^ in CH_2_Cl_2_ and its TDDFT‐computed spectrum. b) UV/Vis/NIR spectrum of QH_2_^+^ obtained by treatment of SQH_2_^.^ with an excess of NOSbF_6_ and its TDDFT‐computed spectrum. Calculated data were redshifted by 24 and 54 nm, respectively. The data sets were normalized to the maximum of the absorption bands at *λ*=397 and 427 nm, respectively. c) Main participating orbitals of the three lowest‐energy vertical electronic transitions (TD‐UB3LYP‐D3(BJ)/def2‐TZVPP; threshold: 70 %). The α‐HOMO can be interpreted as the SOMO of the molecule.

After optimization of the aforementioned synthetic conditions for the preparation of SQH_2_^.^, we further examined its spectroscopic and electrochemical properties. SQH_2_^.^ shows a triplet signal in the solution EPR spectrum (Figure 3 a) due to spin coupling of the unpaired electron with the central ^14^N nucleus (*g* ^*iso*^=2.00332, *A* ^*iso*^=672 μT). Further couplings to the outer two nitrogen atoms or protons were not resolved. During the oxidation of [CatH_5_][Br]_2_ under nonbasic conditions, metastable SQH_3_^.+^ was observed by means of EPR spectroscopy (Figure 3 b). It shows additional coupling to the nitrogen‐bound hydrogen nucleus. Simulation of the EPR spectra of both species reproduced the experimental findings and confirmed the absence of a proton directly attached to the aminyl nitrogen in SQH_2_^.^ (Figure 3 a and b). By employing the McConnell equation, *A* ^*iso*^=*Qρ*,^14^ the spin density at the central nitrogen atom in SQH_2_^.^ was estimated to be *ρ_N_*=0.407, if *Q*=1.65 mT was assumed.^15^ This is in reasonable agreement with a value of *ρ_N_*=0.340 obtained from a natural bond orbital (NBO) population analysis at the UTPSS‐D3(BJ)/def2‐TZVPP level of theory (Figure 3 c). In contrast, for N‐protonated SQH_3_^.+^, the aminyl nitrogen spin density was calculated to be only 0.248, which was in line with previous EPR spectroscopy studies.^15^ *A* ^*iso*^/*A* ^0^ gave a value close to 1 % for the amount of s character of the SOMO (*A* ^0^=55.2 mT).^16^ Thus, the aminyl nitrogen contributes almost entirely to the SOMO of the molecule with its 2 p_*z*_ orbital, and thus, SQH_2_^.^ is characterized as a π‐type aminyl radical (Figure 4 c).11i

In the UV/Vis/NIR spectrum of SQH_2_^.^, three absorption bands were found in the UV regime (*λ*=397, 319, and 246 nm). Broad bands in the visible region at *λ*=539 and 675 nm (shoulder at *λ*=776 nm) account for the dark‐blue color (Figure 4 a). TDDFT calculations at the TD‐UB3LYP‐D3(BJ)/def2‐TZVPP level of theory are in excellent agreement with the experimental data and identified the respective electronic transitions (Figure 4 b). The UV/Vis spectrum of fully oxidized QH_2_^+^ (obtained by the addition of NOSbF_6_) has distinct absorption bands in the UV region (Figure 4 b) at *λ*=323, 354, 427, and 770 nm, which are again in nice agreement with the computed absorption spectrum. Importantly, upon bubbling pure dioxygen gas through a solution of SQH_2_^.^ in CH_2_Cl_2_ for 15 min, no changes or the occurrence of absorption bands corresponding to QH_2_^+^ were observed. This finding underscores the pronounced stability of the neutral aminyl radical SQH_2_^.^ against air in nonacidic solution. Notably, upon acidification of the solution with HCl, SQH_2_^.^ is readily oxidized to QH_2_^+^ upon exposure to air.

**Figure 5:**
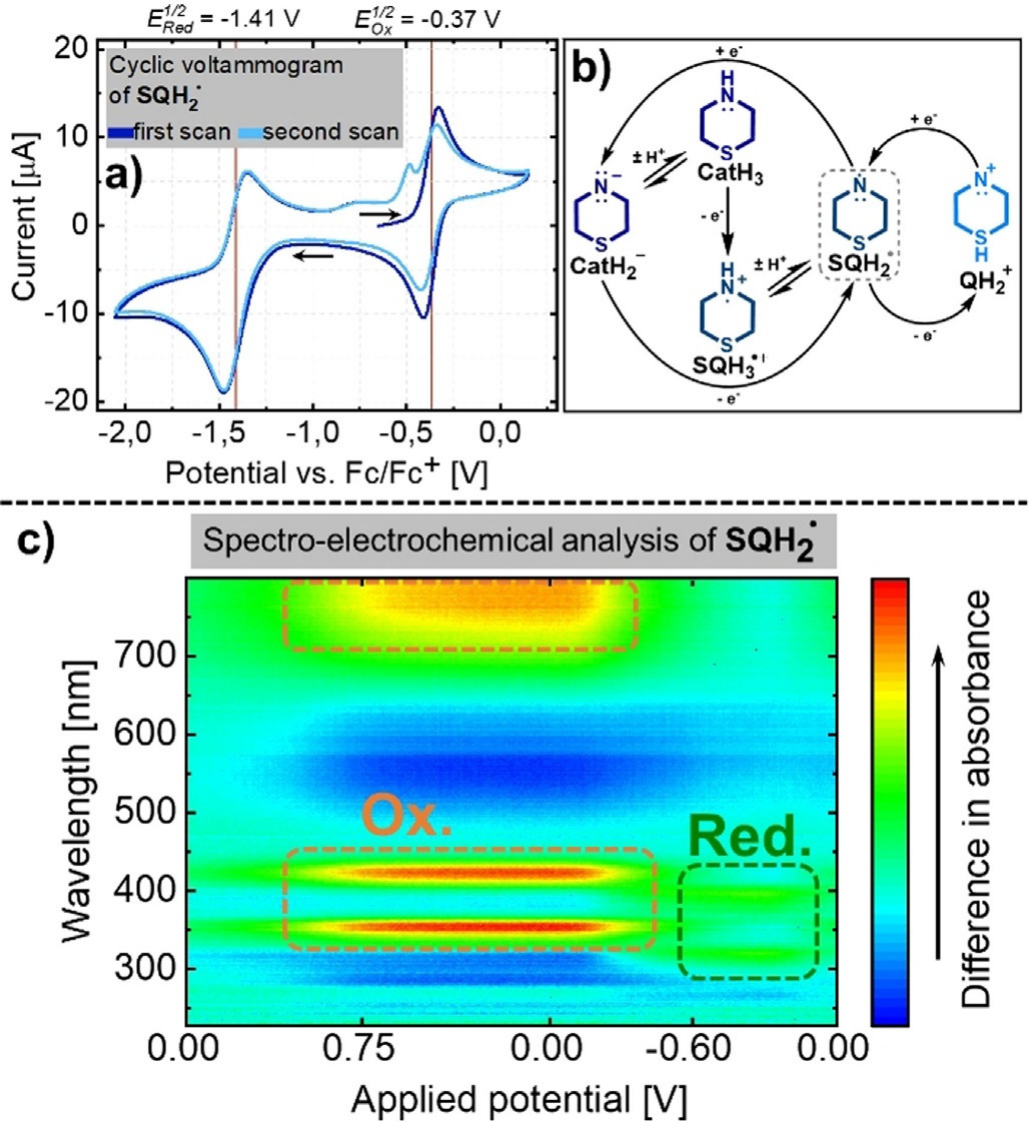
a) Cyclic voltammogram of SQH_2_^.^ in CH_2_Cl_2_. Fc/Fc^+^=ferrocene/ferrocenium couple. b) Possible rationale for the observed cyclic voltammogram. The middle ring of the phenothiazine moiety schematically represents the three different oxidation states. c) Coupled cyclic voltammogram–UV/Vis/NIR absorption spectrum of SQH_2_^.^ in CH_2_Cl_2_. The UV/Vis/NIR absorption spectrum is referenced against a solution containing supporting electrolyte and analyte.

The cyclic voltammogram of SQH_2_^.^ was recorded in CH_2_Cl_2_ (Figure 5 a). In the first scan, a Nernstian one‐electron oxidation (peak current ratio of 0.97) was observed with a half‐wave potential of *E*_1/2_=−0.37 V (vs. Fc/Fc^+^), proposed as the redox couple SQH_2_^.^/QH_2_^+^ (see below). In the cathodic direction, one‐electron reduction with *E*_1/2_=−1.41 V (vs. Fc/Fc^+^) was found for the redox couple SQH_2_^.^/CatH_2_^−^. A peak current ratio of only 0.74 revealed that this redox event was not fully reversible. Indeed, a new oxidation wave appeared during the second scan. This may be explained by a partial protonation of the reduced catechol‐type species, CatH_2_^−^, to form CatH_3_, which is then oxidized to the radical cation, SQH_3_^.+^, at around −0.53 V (Figure 5 b). As a source of protons, CH_2_Cl_2_ as the solvent or other analyte molecules may be considered. Spectro‐electrochemical analysis of SQH_2_^.^ allowed for the potential‐resolved UV/Vis/NIR spectroscopic characterization of the species involved (Figure 5 c). Increasing oxidative potential gave rise to intense absorption bands, with maximum wavelengths of around *λ*=350, 420, and 780 nm. This is in agreement with the absorption spectrum obtained for the sample of QH_2_^+^ (see Figure 4 b). Therefore, the redox couple SQH_2_^.^/QH_2_^+^ can be clearly assigned to the observed reversible oxidation (*E*_1/2_=−0.37 V). In the cathodic direction, new maxima at *λ*=322 and 414 nm appeared, whereas the transitions in the visible‐light region vanished. This spectrum most likely belongs to the much less intensely colored reduced states CatH_2_^−^ or CatH_3_.

After we had characterized SQH_2_^.^ spectroscopically and electrochemically, we further studied the stability of the radical. Computationally, the radical stabilization energy (RSE) of aminyl radicals can be quantified as the difference in gas‐phase N−H bond dissociation enthalpy (BDE) with NH_3_ as a reference (RSE(SQH_2_^.^)=BDE(CatH_3_)−BDE(NH_3_).^17^ Calculations at the UB3LYP‐D3(BJ)/6‐31G(d) level of theory gave a RSE of −169.4 kJ mol^−1^ for SQH_2_^.^. For comparison, the RSE for the parent phenothiazin‐10‐yl radical (−122.4 kJ mol^−1^) and the 3,7‐dimethoxy‐substituted phenothiazin‐10‐yl radical (−136.7 kJ mol^−1^) were computed. Both values are in excellent agreement with their experimentally derived gas‐phase data (−118.3 and −131.2 kJ mol^−1^, respectively),10j which strongly supports the theoretical method used. Thus, the phenothiazine building block provides most of the stabilizing influence. However, the substituents in SQH_2_^.^ not only stabilize the open‐shell state kinetically, but also have a strong thermodynamic effect that can be attributed to the resonance of the two secondary amine nitrogen atoms. It represents a degree of stabilization that is unrivaled by essentially every other non‐heteroatom‐substituted nitrogen radical and accounts for the experimentally observed stability.^18^ This very large RSE for SQH_2_^.^ encouraged us to address the N−H bond strength experimentally. SQH_2_^.^ was subjected to sterically nonhindered hydrogen‐atom donors with known BDE(E‐H) and the reactions were followed spectroscopically. No reaction occurred with compounds down to a BDE of 319 kJ mol^−1^ (phenols, silane, catechols, ethane thiol, 9,10‐dihydroanthracene), but was observed only with potent hydrogen‐atom donors, such as TEMPO‐H (TEMPO=(2,2,6,6‐tetramethylpiperidin‐1‐yl)oxyl) or triphenylstannane (for a full table of comparison, see the Supporting Information). This series of measurements allowed the BDE of CatH_3_ in CH_2_Cl_2_ to be bracketed between 319 and 297 kJ mol^−1^, which was in reasonable agreement with the computational results in the gas phase (281 kJ mol^−1^).

## Conclusions

SQH_2_^.^ represents the first stable N‐unsubstituted phenothiazinyl radical and extends the limited class of stable free aminyl radicals.^11^ By means of EPR spectroscopy, the spin density at the aminyl nitrogen atom was estimated to be 0.401. The UV/Vis/NIR absorption spectrum of SQH_2_^.^ showed three relatively sharp absorption bands in the UV regime, as well as two broad bands in the visible region, which rationalized the deep dark‐blue color of the compound. One‐electron oxidation and reduction of SQH_2_^.^ was observed at *E*_1/2_=−0.37 and −1.41 V (vs. Fc/Fc^+^), respectively. The RSE was calculated to be −169.4 kJ mol^−1^ and supported experimentally. Thus, SQH_2_^.^ is one of the most stable non‐heteroatom‐substituted nitrogen radicals and, in turn, the N−H bond in the parent CatH_3_ is one of the weakest ever determined. The high radical stability is attributed to extensive spin delocalization over the phenothiazine backbone and the two external amino nitrogen atoms. Radical persistency is provided by steric shielding of reactive positions. At first, this compound might serve as a reference species for the field of phenothiazine‐derived drugs or dye stuffs. Moreover, the tridentate ligand nature makes this compound ideally suited for future investigations within the field of redox‐active and/or structural‐strain‐imposing ligands.^19^ The proximity of protic hydrogen atoms next to the radical center equips SQH_2_^.^ with the ideal ingredients for proton‐coupled electron transfer. Finally, the remarkable lifetime of SQH_2_^.^ promises applications in vibrant areas of research such as electron relay shuttling,^20^ side‐directed spin‐labeling,^21^ or as a building block for organic magnetic materials.^22^ Several of these aspects are under current investigation.

## Conflict of interest

The authors declare no conflict of interest.

## Supporting information

As a service to our authors and readers, this journal provides supporting information supplied by the authors. Such materials are peer reviewed and may be re‐organized for online delivery, but are not copy‐edited or typeset. Technical support issues arising from supporting information (other than missing files) should be addressed to the authors.

Supplementary
