# Supplementary material for: An Air‐Stable, Neutral Phenothiazinyl Radical with Substantial Radical Stabilization Energy
Source: Chemistry. 2020 Feb 19;26(14):3152–6. doi: 10.1002/chem.201905238 (PMC7079145; doi:10.1002/chem.201905238)
Supplement: Supplementary file 1 — Supplementary [file CHEM-26-3152-s001.pdf]

CHEMISTRY
A European Journal

Supporting Information

An Air-Stable, Neutral Phenothiazinyl Radical with Substantial Radical Stabilization Energy

Lukas M. Sigmund+, Fabian Ebner+, Christoph Jöst, Jonas Spengler, Nils Gönnheimer, Deborah Hartmann, and Lutz Greb*[a]

chem_201905238_sm_miscellaneous_information.pdf

Table of Contents

1 Experimental Details 1

1.1 Synthetic Procedures and Characterization Data 1

1.2 Reactivity Studies 8

1.3 NMR Spectroscopy 9

1.4 Evans NMR Study 19

1.5 EPR Spectroscopy 20

1.6 IR Spectra 21

1.7 UV-Vis/NIR Spectroscopy 22

1.8 Mass Spectrometry 22

1.9 Cyclic Voltammetry and Spectro-electrochemical Analysis 23

1.10 X-Ray diffraction analyses 23

2 Computational Details 27

2.1 Radical Stabilization Energy 28

2.2 xyz Coordinates 28

3 References 44

1 Experimental Details

All reagents and solvents were purchased from commercial sources and were used as received unless otherwise noted. All used solvents were degassed prior to use either by three freeze-pump-thaw cycles or by ultrasonication with short vacuum application and were stored over activated molecular sieve (3 or 4 Å) under dry argon. All reactions were carried out in flame-dried standard laboratory glassware under a dry argon atmosphere using Schlenk line techniques and were permanently magnetically stirred. All syringes, magnetic stirring bars, needles, and transfer cannulas were dried and/or flushed with argon prior to use. Compounds sensitive to ambient conditions were stored and handled in a glove box (MBraun LABmaster dp, MB-20-G) filled with dry nitrogen gas. Removal of solvents or other volatiles *in vacuo* was performed using a Heidolph VV2000 rotary evaporator or a Schlenk line. Reported yields refer to isolated yields of analytically pure material and are the result of one specific reaction. Compounds known to literature were synthesized following published procedures, which are cited. Analytical data of literature-known compounds were compared to data of the respective reference and were found to be consistent in all cases. Novel compounds were characterized to the reported structure to best of our knowledge.

1.1 Synthetic Procedures and Characterization Data

3,7-Di-*tert*-butyl-10*H*-phenothiazine (**2**)[1]

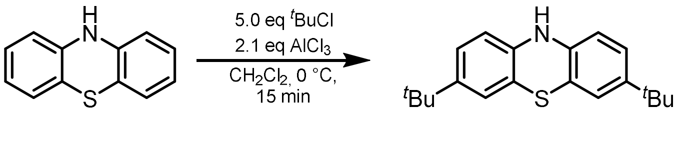

1
199.27 g/mol

2
311.49 g/mol

In a two-necked Schlenk flask with overpressure valve and dropping funnel phenothiazine **1** (20.0 g, 100.4 mmol, 1.0 eq) and anhydrous $AlCl_3$ (28.1 g, 210.7 mmol, 2.1 eq) were suspended in anhydrous dichloromethane (200 mL). 2-Chloro-2-methylpropane (55.0 mL, 46.2 g, 499.1 mmol, 5.0 eq) was added dropwise to the stirred suspension at 0 °C. After the addition, the reaction mixture was stirred for 15 min at 0 °C. Then, the red mixture was poured into distilled water (1.0 L) and it was neutralized by adding sodium hydrogen carbonate (60 g). Subsequently, the phases were separated, and the aqueous phase was extracted with dichloromethane (3 × 200 mL). The combined organic phases were washed with saturated sodium dithionite solution. The color of the solution turned from red to yellow. It was dried over sodium sulfate. It was filtered, and the solvent was removed under reduced pressure. The crude product was washed with *n*-hexane and

dried *in vacuo*. 3,7-Di-*tert*-butyl-10*H*-phenothiazine **2** was obtained as a colorless solid (18.2 g, 58.5 mmol, 58%) that slowly turns red upon exposure to air.

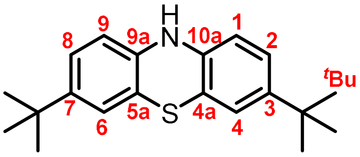

^{1}**H NMR** (400 MHz, CD_2Cl_2): δ [ppm] = **7.03-7.00** (m, 4H, H-1/2/8/9), **6.55-6.50** (m, 2H, H-4/6), **5.81** (br s, 1H, NH), **1.24** (s, 18H, ^{t}Bu).

HR-MS (positive ion DART): *m/z* calculated for $C_{20}H_{26}NS^+$ [M+H]$^+$: 312.1780, found: **312.1774**.

1,9-Dibromo-3,7-di-*tert*-butyl-10*H*-phenothiazine (3)[2]

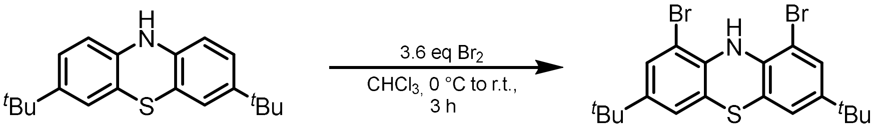

2
311.49 g/mol

3
469.28 g/mol

In a flame-dried and argon filled Schlenk flask with a dropping funnel, 3,7-di-*tert*-butyl-10*H*-phenothiazine **2** (5.0 g, 16.1 mmol, 1.0 eq) was dissolved in anhydrous and degassed chloroform (46 mL). The dark-red solution was cooled down to 0 °C and bromine (3.0 mL, 9.4 g, 58.6 mmol, 3.6 eq) was added dropwise within 10 min. It was stirred for 2.5 h at room temperature. The resulting black mixture was diluted with chloroform (20 mL). The reaction was quenched with hydrazine monohydride (6.0 mL, 6.2 g, 123.7 mmol) and saturated sodium carbonate solution (100 mL) was added. The phases were separated, and the aqueous phase was extracted with dichloromethane (2 × 50 mL). The combined organic phases were washed with brine (2 × 100 mL) and dried over sodium sulfate. It was filtered, and the solvent was evaporated under reduced pressure. After drying *in vacuo*, the desired product 1,9-dibromo-3,7-di-*tert*-butyl-10*H*-phenothiazine **3** (5.29 g, 11.3 mmol, 70%) was obtained as an off-white solid which slowly decomposes upon exposure to air.

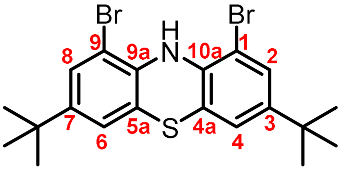

^{1}**H NMR** (400 MHz, CD_2Cl_2): δ [ppm] = **7.28** (d, $^4J_{HH}$ = 2.0 Hz, 2H, H-2/8), **7.07** (br s, 1H, NH), **6.98** (d, $^4J_{HH}$ = 2.0 Hz, 2H, H-4/6), **1.24** (s, 18H, ^{t}Bu).

^{13}C{^{1}H} NMR (100 MHz, CD_2Cl_2): δ [ppm] = **147.6** (C_q, C-3/7), **136.7** (C_q, C-9a/10a), **128.2** (CH, C-2/8), **123.3** (CH, C-4/6), **119.7** (C_q, C-1/9), **110.0** (C_q, C-4a/5a), **34.6** (C_q, ^{t}Bu), **31.2** (CH_3, ^{t}Bu).

HR-MS (positive ion DART): *m/z* calculated for $C_{20}H_{24}Br_2NS^+$ $[M+H]^+$ 469.9970, found **469.9971**.

10-Benzyl-1,9-dibromo-3,7-di-*tert*-butyl-10*H*-phenothiazine (**4**)[3]

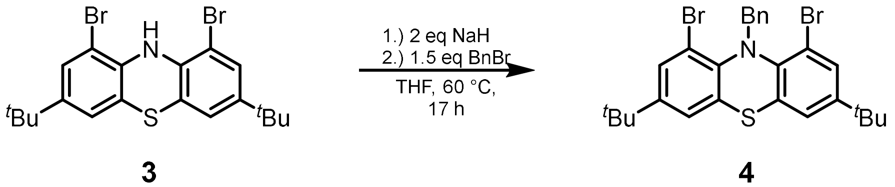

3
469.28 g/mol

4
559.40 g/mol

A flame-dried and argon filled Schlenk flask with overpressure valve was charged with 1,9-dibromo-3,7-di-*tert*-butyl-10*H*-phenothiazine **3** (5.3 g, 11.3 mmol, 1.0 eq) and sodium hydride (547 mg, 22.8 mmol, 2.0 eq). Anhydrous and degassed THF (46 mL) was added. The resulting dark-green mixture was heated to reflux for 1 h. It was cooled down to room temperature and benzyl bromide (2.0 mL, 2.88 g, 16.8 mmol, 1.5 eq) was added dropwise within 5 min. The reaction mixture was stirred at 60 °C for 17 h and subsequently was cooled down to room temperature. Hydrochloric acid (1 M, 25 mL, 25.0 mmol, 2.2 eq) was added to the dark-yellow mixture. The color changed to dark-red. Diethyl ether (50 mL) was added and the phases were separated. The aqueous phase was extracted with diethyl ether (2 × 25 mL). The combined organic phases were washed with brine (2 × 30 mL) and dried over sodium sulfate. It was filtered, and the solvent was removed under reduced pressure. To the obtained orange-brown oil, ethanol (20 mL) was added and the crude product was crystallized over night at -20 °C. The precipitate was separated, washed with ethanol (25 mL) and dried *in vacuo*. The target compound 10-benzyl-1,9-dibromo-3,7-di-*tert*-butyl-10*H*-phenothiazine **4** (5.0 g, 8.9 mmol, 79%) was obtained as a slightly orange solid, which showed stability against ambient conditions.

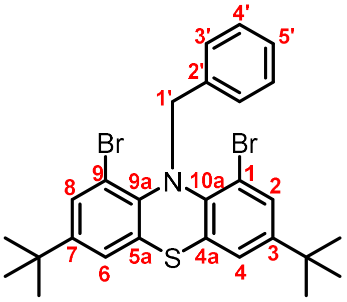

^{1}H NMR (400 MHz, CD_2Cl_2): δ [ppm] = **7.49** (d, $^{4}J_{HH}$ = 2.09 Hz, 2H, H-2/8), **7.21-7.16** (m, 1H, H-5'), **7.12-7.08** (m, 2H, H-4'), **7.04** (d, $^{4}J_{HH}$ = 2.11 Hz, 2H, H-4/6), **7.00-6.97** (m, 2H, H-3'), **4.82** (s, 2H, H-1'), **1.27** (s, 18H, ^{t}Bu).

^{13}C{^{1}H} NMR (100 MHz, CD_2Cl_2): δ [ppm] = **150.3** (C_q, C-3/7), **140.0** (C_q, C-9a/10a), **138.3** (C_q, C-2'), **136.1** (C_q, C-1/9), **129.8** (CH, C-3'), **129.6** (CH, C-2/8), **128.2** (CH, C-4'), **127.7** (CH, C-5'), **123.6** (CH, C-4/6), **120.31** (C_q, C-4a/5a), **59.1** (CH_2, C-1'), **34.8** (C_q, ^{t}Bu), **31.2** (CH_3, ^{t}Bu).

HR-MS (positive ion DART): *m/z* calculated for $C_{27}H_{30}Br_2NS^+$ [M+H]$^+$ 560.0440, found **560.0451**.

10-Benzyl-3,7-di-*tert*-butyl-*N*^{1},*N*^{9}-bis(2,6-diisopropylphenyl)-10*H*-phenothiazine-1,9-diamine (**5**)

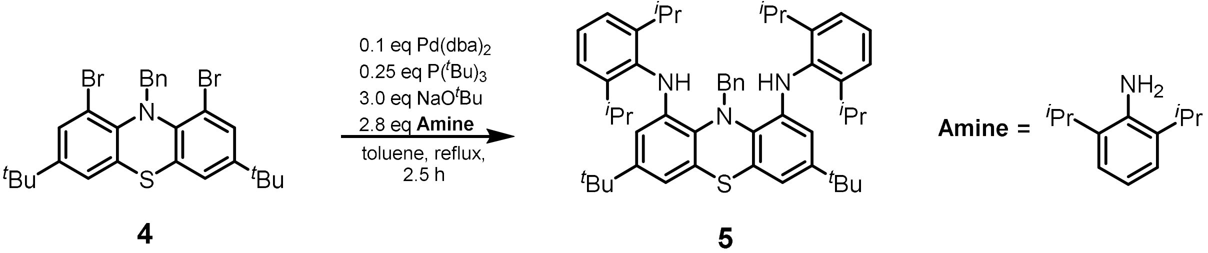

4

559.40 g/mol

5

752.16 g/mol

177.29 g/mol

A flame-dried and argon filled Schlenk flask with check/overpressure valve was charged with 10-benzyl-1,9-dibromo-3,7-di-*tert*-butyl-10*H*-phenothiazine **4** (1.5 g, 2.68 mmol, 1.0 eq), bis(dibenzylideneacetone)palladium(0) (154 mg, 0.27 mmol, 0.1 eq), tri-*tert*-butylphosphane (135 mg, 0.67, 0.25 eq) and sodium *tert*-butoxide (780 mg, 8.11 mmol, 3.0 eq). Anhydrous and degassed toluene (30 mL) was added. To the stirred mixture, 2,6-diisopropylaniline (1316 mg, 7.42 mmol, 2.8 eq) was added, and it was heated to reflux for 2.5 h. The reaction mixture was allowed to cool down to room temperature and was filtered through a pad of celite. After filtration, the pad was rinsed with ethyl acetate. The dark-green solution was washed with water, saturated sodium dithionite solution and brine (each 20 mL). The combined aqueous phases were extracted with ethyl

acetate (1 × 15 mL). The obtained organic phase was washed with brine (2 × 15 mL). The combined organic phases were dried over sodium sulfate. It was filtered, and the solvent was removed under reduced pressure. The obtained black oil was washed with pentane (15 mL) and the desired product 10-benzyl-3,7-di-*tert*-butyl-N^1,N^9-bis(2,6-diisopropylphenyl)-10*H*-phenothiazine-1,9-diamine **5** (870 mg, 1.16 mmol, 43%) was obtained as a slightly green powder after several recrystallization steps from ethanol and drying *in vacuo*.

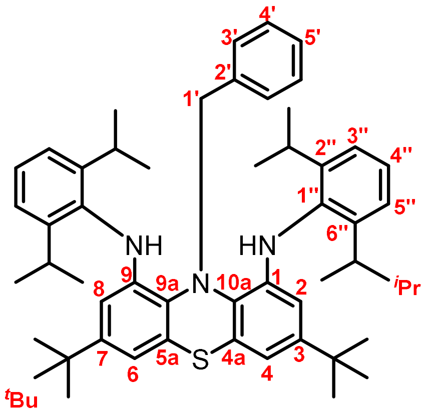

^{1}H NMR (400 MHz, $CDCl_3$): δ [ppm] = **7.36-7.31** (m, 4H, H-3''/5''), **7.30-7.28** (m, 2H, H-5'), **7.29-7.23** (m, 1H, H-4''), **7.17** (t, $^4J_{HH}$ = 7.6 Hz 2H, H-4'), **6.77-6.81** (m, 2H, H-3'), **6.41** (d, $^4J_{HH}$ = 2.1 Hz, 2H, H-4/6), **6.05** (d, $^4J_{HH}$ = 2.1 Hz, 2H, H-2/8), **5.78** (s, 2H, NH), **4.65** (s, 2H, H-1'), **3.42** (sept, $^3J_{HH}$ = 6.9 Hz, 2H, ^{i}Pr), **3.11** (sept, $^3J_{HH}$ = 6.9 Hz, 2H, ^{i}Pr), **1.25** (d, $^3J_{HH}$ = 7.0 Hz, 6H, ^{i}Pr), **1.23** (d, $^3J_{HH}$ = 7.0 Hz, 6H, ^{i}Pr), **1.11** (s, 18H, ^{t}Bu), **1.05** (d, $^3J_{HH}$ = 6.9 Hz, 6H, ^{i}Pr).

^{13}C{^{1}H} NMR (100 MHz, $CDCl_3$): δ [ppm] = **149.2** (C_q, C-3/7), **148.2** (C_q, C-2'' or C-6''), **147.2** (C_q, C-2'' or C-6''), **142.0** (C_q, C-4a/5a), **137.2** (C_q, C-2'), **135.0** (C_q, C-1''), **133.4** (C_q, C-1/9), **130.3** (CH, C-3'), **128.1** (CH, C-4'), **127.6** (CH, C-3'' or C-5''), **127.5** (CH, C-3'' or C-5''), **125.7** (C_q, C-9a/10a), **124.1** (CH, C-5'), **124.0** (CH, C-4''), **112.5** (CH, C-4/6), **108.1** (CH, C-2/8), **54.8** (CH_2, C-1'), **34.6** (C_q, ^{t}Bu), **31.3** (CH_3, ^{t}Bu), **28.7** (CH, ^{i}Pr), **28.4** (CH, ^{i}Pr), **24.7** (CH_3, ^{i}Pr), **24.32** (CH_3, ^{i}Pr), **24.31** (CH_3, ^{i}Pr), **23.4** (CH_3, ^{i}Pr).

HR-MS (positive ion DART): *m*/*z* calculated for $C_{51}H_{66}N_3S^+$ $[M+H]^+$ 752.4972, found **752.4975**.

3,7-Di-*tert*-butyl-N^1,N^9-bis(2,6-diisopropylphenyl)-10*H*-phenothiazine-1,9-diaminium bromide ([**CatH$_5$**]**Br$_2$**)

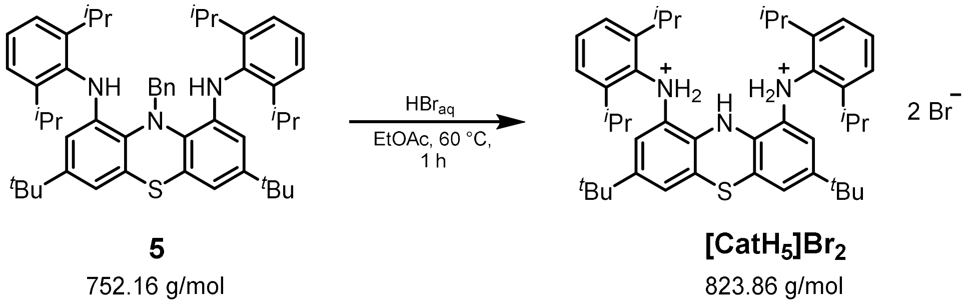

In a flame-dried and argon filled Schlenk flask 10-benzyl-3,7-di-*tert*-butyl-N^1,N^9-bis(2,6-diisopropylphenyl)-10*H*-phenothiazine-1,9-diamine **5** (207 mg, 0.28 mmol, 1.0 eq) was dissolved in degassed ethyl acetate (8 mL). To the stirred pale green solution concentrated hydrobromic acid (8.8 M, 180 µL, 1.58 mmol, 5.6 eq) was added within 1 min. Immediately, the color changed to golden brown. The solution was heated to 60 °C for 1 h. The dark-brown solution was allowed to cool down to room temperature and all volatiles were removed under reduced pressure. Under nitrogen, the obtained solid was washed with hexane (6 × 3 mL) to give an orange solid, which was characterized to the desired product 3,7-di-*tert*-butyl-N^1,N^9-bis(2,6-diisopropylphenyl)-10*H*-phenothiazine-1,9-diaminium bromide **6** (84 mg, 0.10 mmol, 41%). **6** turns green upon exposure to air.

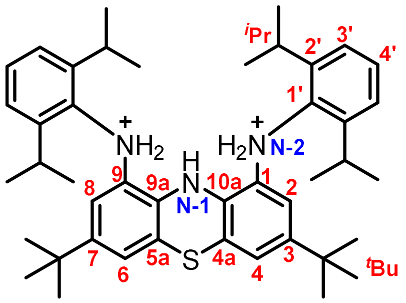

^{1}H NMR (400 MHz, CD_2Cl_2): δ [ppm] = **12.32** (s, 4H, NH-2), **9.94** (s, 1H, NH-1), **7.55** (t, 2H, $^3J_{HH}$ = 7.8 Hz, 2H, H-4'), **7.40** (d, $^3J_{HH}$ = 7.8 Hz, 4H, H-3'), **7.09** (s, 2H, H-2/8), **6.24** (s, 2H, H-4/6), **3.37** (sept, $^3J_{HH}$ = 6.6 Hz, 4H, ^{i}Pr), **1.39** (d, $^3J_{HH}$ = 6.6 Hz, 12H, ^{i}Pr), **1.05** (s, 18H, ^{t}Bu), **1.03** (d, $^3J_{HH}$ = 6.6 Hz, 12H, ^{i}Pr).

^{13}C{^{1}H} NMR (150 MHz, CD_2Cl_2): δ [ppm] = **146.9** (C_q, C-3/7), **144.9** (C_q, C-2'), **132.8** (C_q), **131.4** (CH, C-4'), **127.2** (C_q), **126.5** (CH, C-3'), **125.2** (CH, C-2/8), **124.1** (C_q) **123.4** (C_q), **119.1** (CH, C-4/6), **34.7** (C_q, ^{t}Bu), **30.9** (CH_3, ^{t}Bu), **29.6** (CH, ^{i}Pr), **25.5** (CH_3, ^{i}Pr), **24.4** (CH_3, ^{i}Pr).

Detailed assignment of the quaternary carbon atoms was not possible due to insufficient 2-D NMR behavior.

IR (ATR-FTIR): $\tilde{\nu}$ [cm^{-1}] = **3284** (*m*, N-H stretching), **3189** (*m*, N-H stretching), **3083** (*m*, N-H stretching), **2961** (*s*, aromatic C-H stretching), **2926** (*m*, aliphatic C-H stretching), **2868** (*m*, aliphatic C-H stretching), **1603** (*m*, aromatic C-C stretching).

Elemental analysis calculated for $C_{44}H_{61}Br_2N_3S$ [%]: C, **64.15**; H, **7.47**; N, **5.10**. Found [%]: C, **63.08**; H, **7.16**; N, **5.23**.

1,9-Diamino-3,7-di-*tert*-butyl-N^1,N^9-bis(2,6-diisopropylphenyl)-10*H*-phenothiazin-10-yl radical (**SQH$_2^{\bullet}$**)

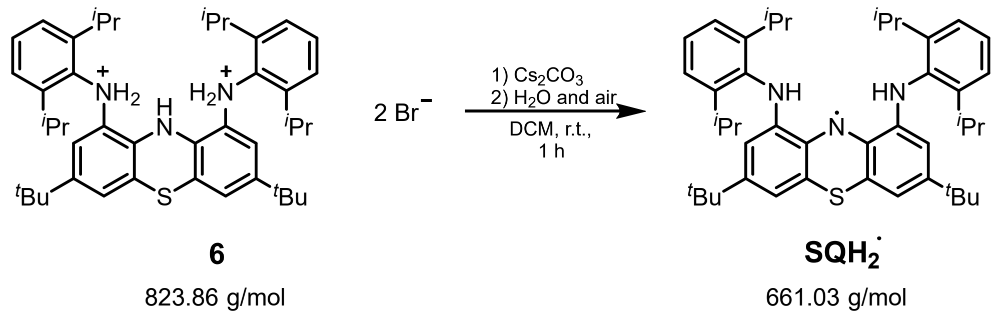

A flame-dried and argon filled Schlenk flask was charged with 3,7-di-*tert*-butyl-N^1,N^9-bis(2,6-diisopropylphenyl)-10*H*-phenothiazine-1,9-diaminium bromide **6** (29 mg, 35.2 µmol, 1.0 eq) and caesium carbonate (73 mg, 224.1 µmol, 6.4 eq). Anhydrous and degassed DCM (3 mL) was added and the orange mixture was stirred for 5 min. Subsequently, water (0.5 mL) was added what caused a change in color to dark green. Air was passed through the solution for 1.5 h. After a few seconds the color changed from green to dark-blue. While oxidizing, a constant solvent volume was maintained. The mixture was washed with water (4 × 1 mL) and the solvent was removed under reduced pressure. The obtained dark-blue solid was dried *in vacuo* to give the 1,9-diamino-3,7-di-*tert*-butyl-N^1,N^9-bis(2,6-diisopropylphenyl)-phenothiazin-10-yl radical **SQH$_2^{\bullet}$** (22 mg, 33.3 µmol, 95%) as an air-stable compound.

EPR (ν_X = 9.448867 GHz, CD_2Cl_2): g^{iso} = 2.002652, triplet with A_N^{iso} = 661 µT.

IR (ATR-FTIR): $\tilde{\nu}$ [cm^{-1}] = **3379** (*m*, symmetric N-H stretching), **3364** (*m*, antisymmetric N-H stretching), **2956** (*s*, aromatic C-H stretching), **2926** (*s*, aliphatic C-H stretching), **2864** (*m*, aliphatic C-H stretching).

Elemental analysis calculated for $C_{44}H_{58}N_3S$ [%]: C, **79.95**; H, **8.84**; N, **6.36**. Found [%]: C, **79.22**; H, **8.86**; N, **6.41**.

1.2 Reactivity Studies

The reactivity of $\mathbf{SQH_2^{\bullet}}$ with potential hydrogen atom donors was studied. This allowed us to narrow down the N-H bond strength with respect to homolytic bond cleavage. All reactions were carried out following the described procedure except otherwise noted.

In a J. Young NMR tube, $\mathbf{SQH_2^{\bullet}}$ (3 mg, 4.5 µmol, 1.0 eq) was dissolved in dry and degassed DCM-*d*2 and the respective substrate was added (22.5 µmol, 5.0 eq). The reaction was followed for several days at room temperature using ^{1}H NMR and EPR spectroscopy.

Table S-1: List of substrates subjected $\boldsymbol{SQH_2^{\bullet}}$, their respective X-H bond dissociation energy (BDE) and the observed reaction. The here specified experimentally determined BDE were taken from ref. [4].

Entry	Substrate	Substrate's X-H BDE [kJ/mol]	Observation
**1**	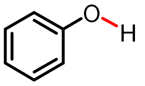 **phenol**	378	No reaction occurred.
**2**	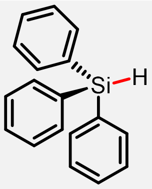 **triphenylsilane**	371	No reaction occurred.
**3**	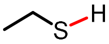 **ethanethiol**	365	No reaction occurred.
**4**	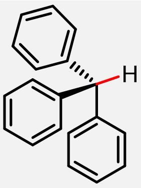 **triphenylmethane**	361	No reaction occurred.
**5**	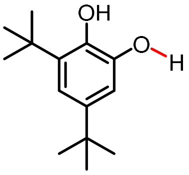 **3,5-di-*tert*-butylcatechol**	332	No reaction occurred.
**6**	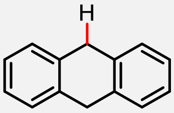 **9,10-dihydroanthracene**	319	No reaction occurred.

Continuation **Table S-1**.

**7**	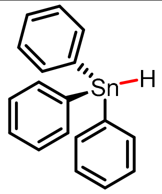 **triphenylstannane**	297	A reaction occurred within minutes. The color of the reaction mixture changed from dark blue to gold-brown. A new triplet-splitted signal arose in the EPR spectrum. $CatH_3$ was detected by NMR spectroscopy.
**8**	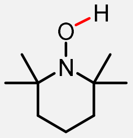 **2,2,6,6-tetramethyl-piperidin-1-ol (TEMPO-H)**	291	A reaction occurred within minutes. The color of the reaction mixture changed from dark-blue to dark-brown. The TEMPO radical was detected by EPR spectroscopy.

1.3 NMR Spectroscopy

All nuclear magnetic resonance spectra were collected on a Bruker Avance II 400 spectrometer at 298 K, except otherwise stated. All synthesized compounds were characterized with ^{1}H and ^{13}C{^{1}H} NMR protocols dissolved in deuterated dichloromethane (CD_2Cl_2) or chloroform ($CDCl_3$) as solvent. Assignment of observed signals was achieved by two-dimensional experiments (heteronuclear multiple-bond correlation spectroscopy, HMBC and heteronuclear single-quantum correlation spectroscopy, HSQC). Chemical shifts δ are given in parts per million (ppm) with reference to tetramethylsilane (TMS). ^{1}H NMR data is reported as follows: chemical shift δ [ppm], multiplicity (s = singlet, br s = broad singlet, d = doublet, t = triplet, quint = quintet, sept = septet, m = multiplet), scalar spin-spin coupling constant [Hz] as $^XJ_{AB}$ (X = number of chemical bonds between coupled nuclei, A, B = coupled nuclei), integration value, signal assignment. ^{1}H NMR spectra are calibrated on solvent residual signals resulting from non-deuterated molecules ($CHDCl_2$ in CD_2Cl_2 at 5.32 ppm, t and $CHCl_3$ in $CDCl_3$ at 7.26, s).[5] ^{13}C{^{1}H} NMR data is reported as follows: chemical shift δ [ppm], type of carbon atom (CH_3, CH_2, CH, C_q), signal assignment. ^{13}C{^{1}H} NMR spectra are calibrated on solvent signals (CD_2Cl_2 at 53.84 ppm, quint and $CDCl_3$ at 77.16, t).[1] Measurements were carried out by the NMR service of the inorganic institute of the University of Heidelberg. In the spectra hereafter solvent signals are marked with a red star (*). NMR spectra were processed and plotted with TopSpin 4.0.7.[6]

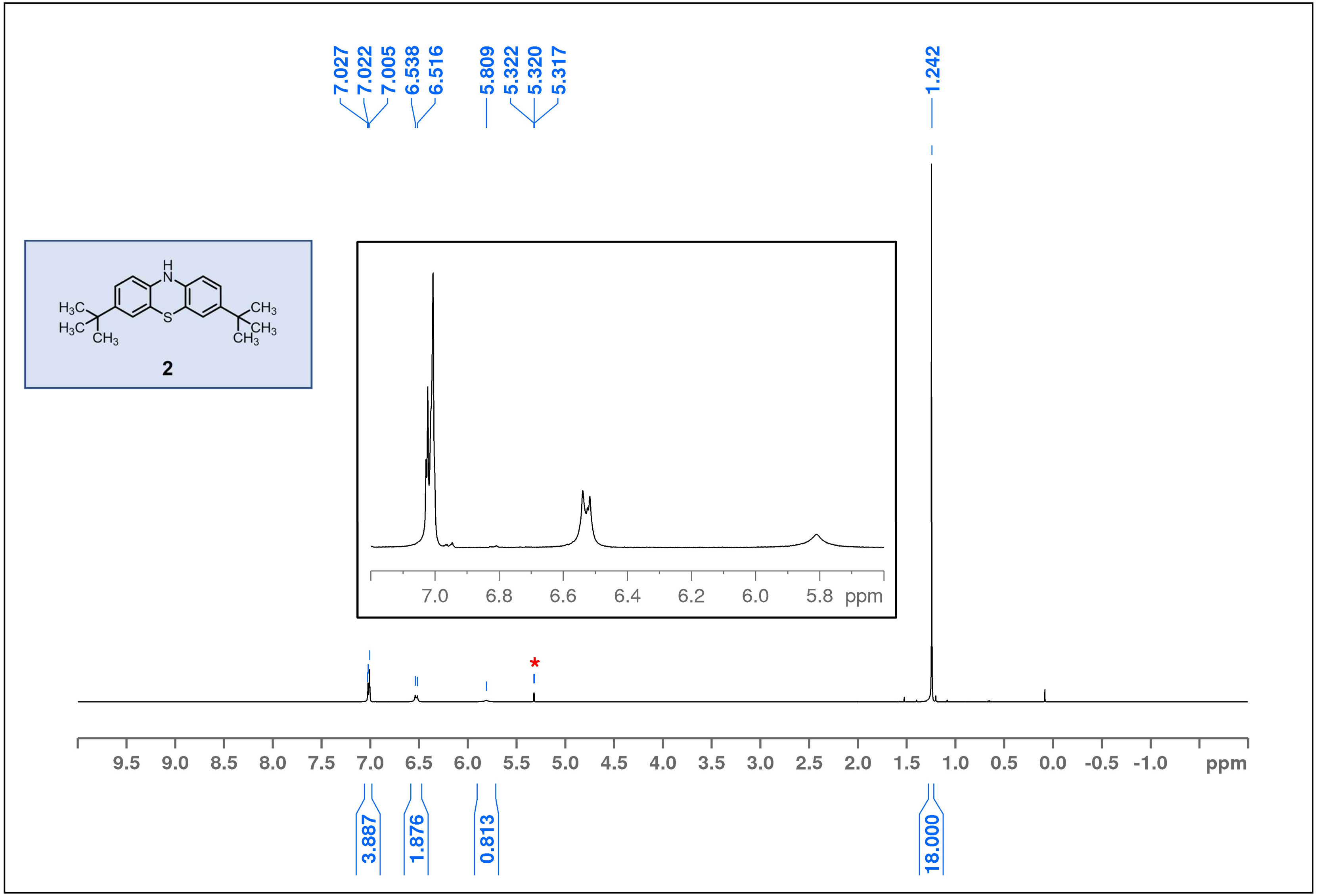

Figure S-1: ^{1}H NMR spectrum of 3,7-di-*tert*-butyl-10*H*-phenothiazine (**2**), 400 MHz, DCM-*d*2, 298 K.

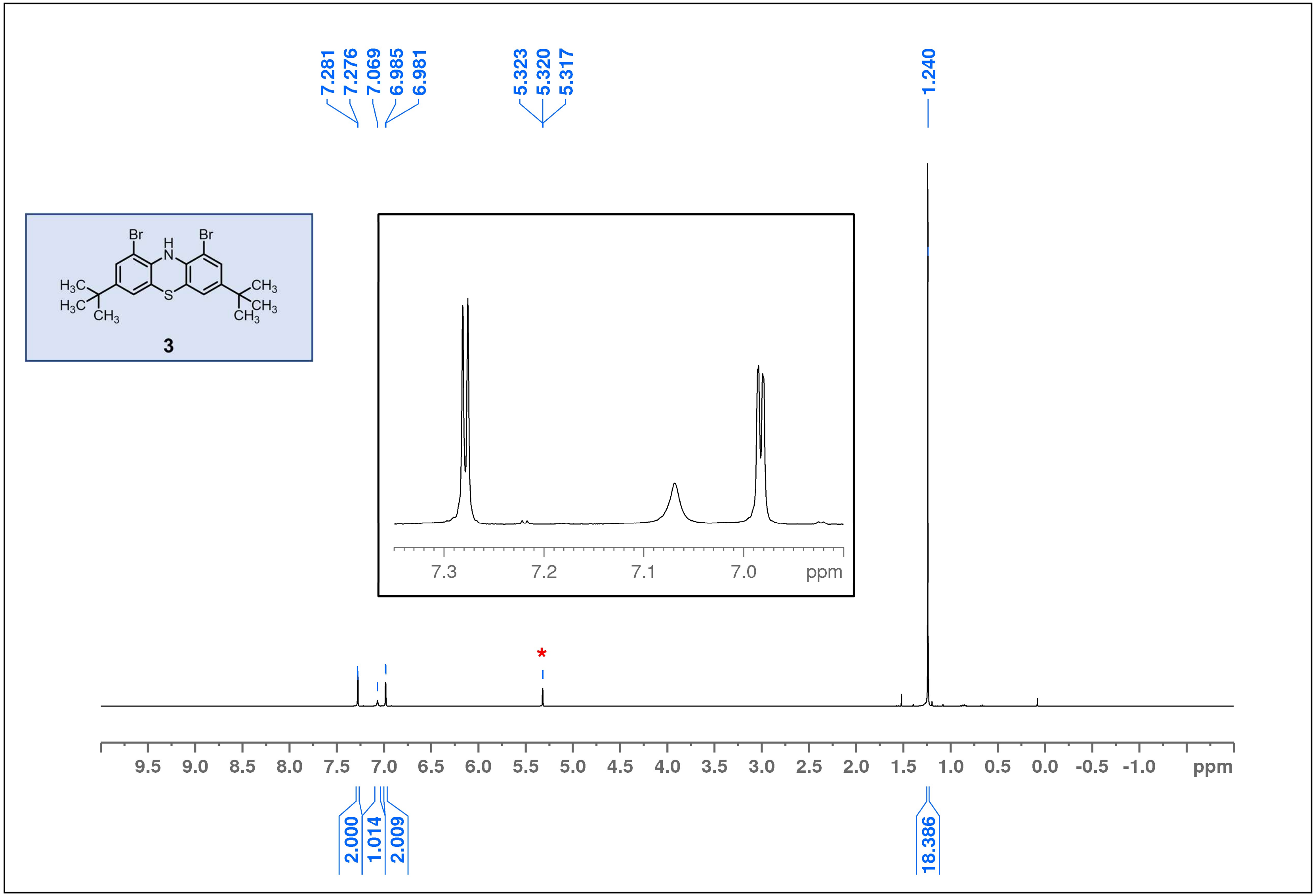

Figure S-2: ^{1}H NMR spectrum of 1,9-dibromo-3,7-di-*tert*-butyl-10*H*-phenothiazine (**3**), 400 MHz, DCM-*d*2, 298 K.

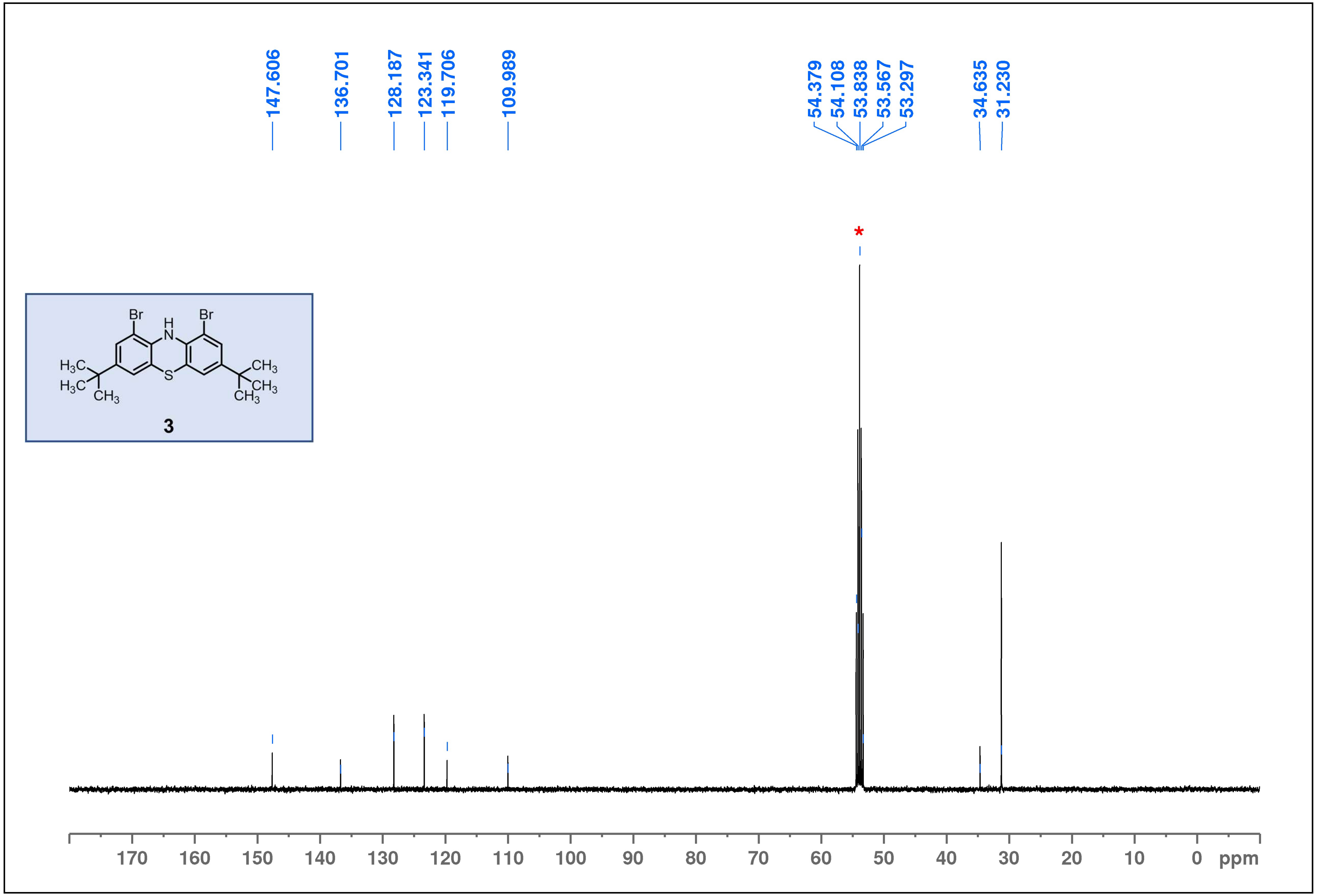

Figure S-3: $^{13}C\{^{1}H\}$ NMR spectrum of ,9-dibromo-3,7-di-*tert*-butyl-10*H*-phenothiazine (**3**), 100 MHz, DCM-*d*2, 298 K.

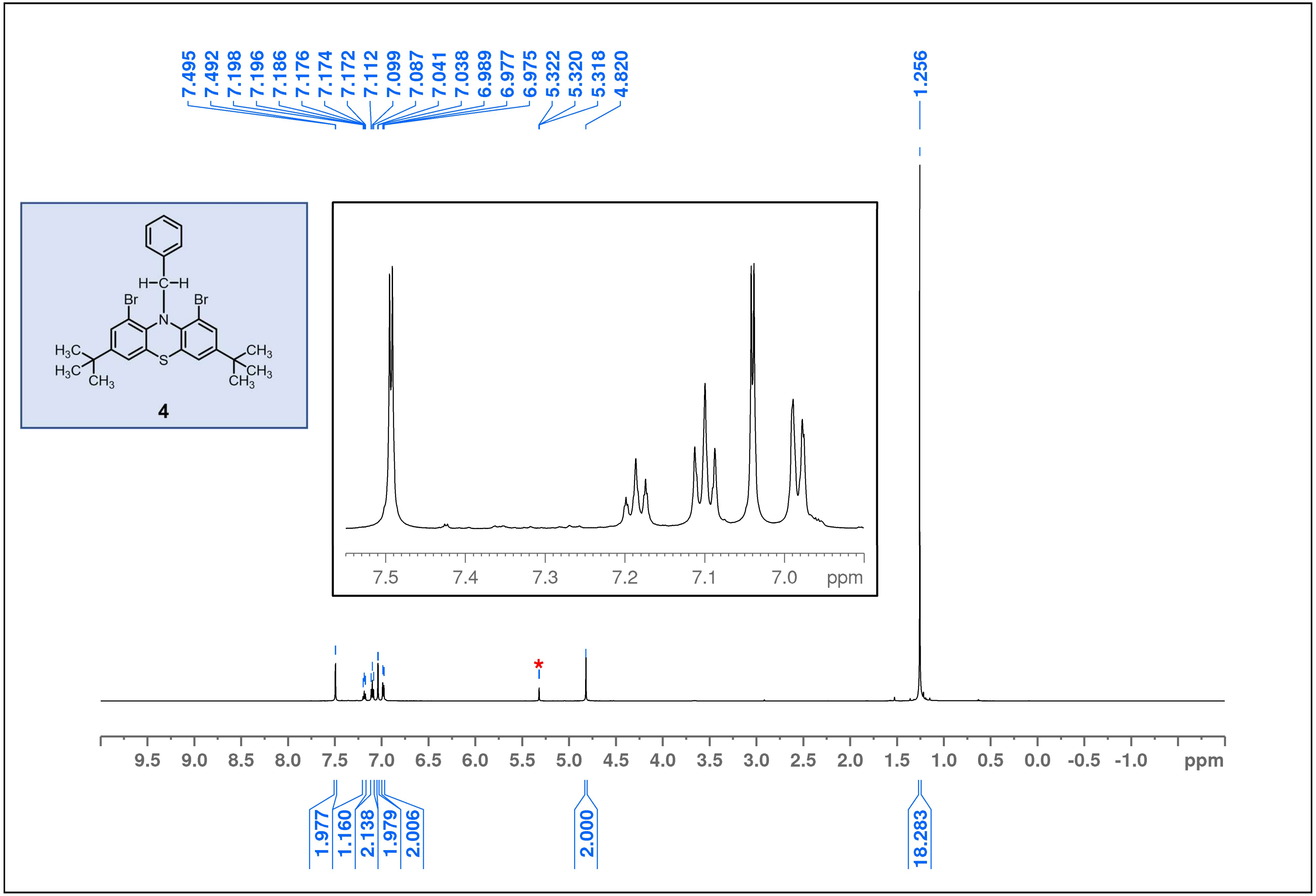

Figure S-4: ^{1}H NMR spectrum of 10-benzyl-1,9-dibromo-3,7-di-*tert*-butyl-10*H*-phenothiazine (**4**), 600 MHz, DCM-*d*2, 298 K.

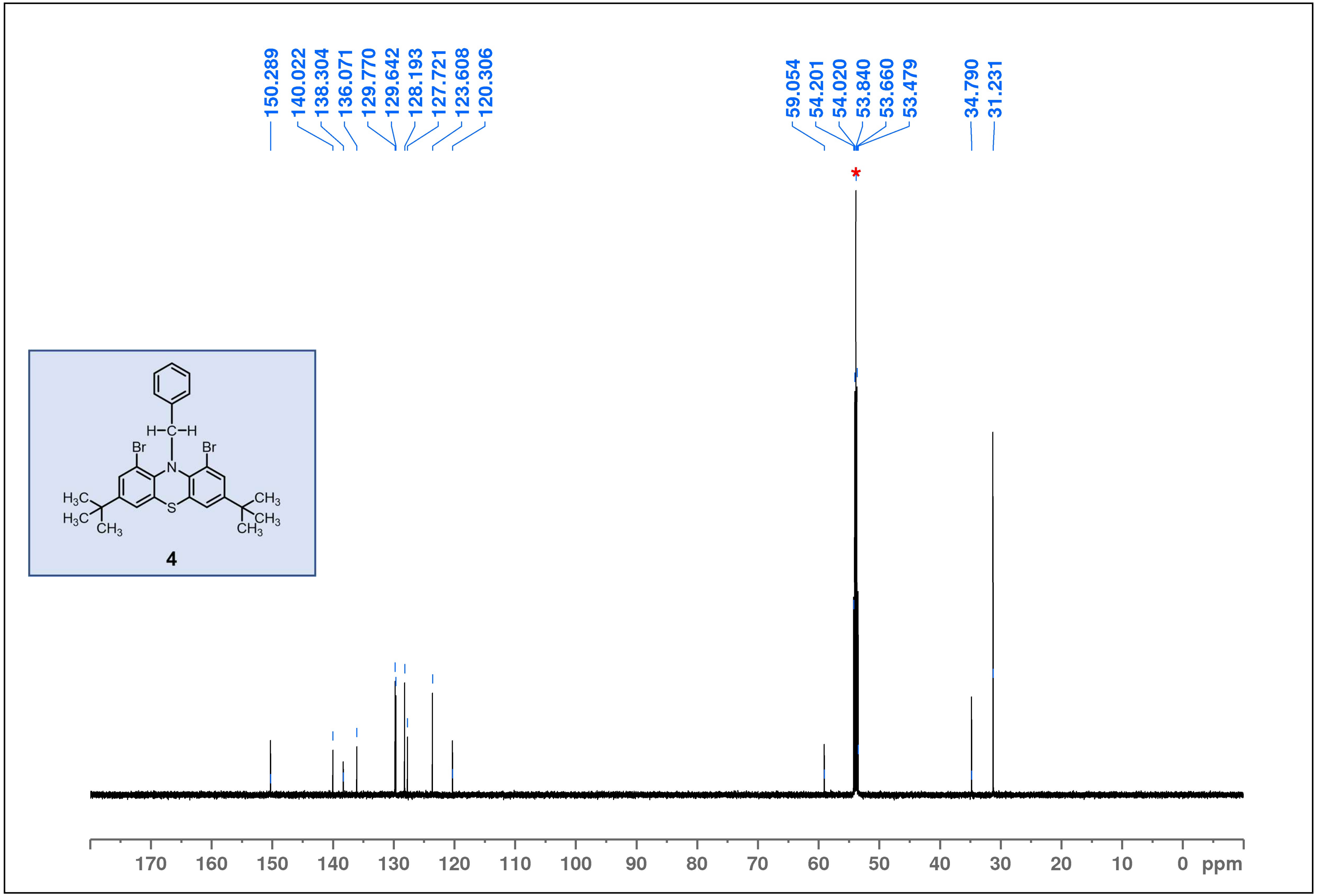

Figure S-5: $^{13}C\{^{1}H\}$ NMR spectrum of 10-benzyl-1,9-dibromo-3,7-di-*tert*-butyl-10*H*-phenothiazine (**4**), 150 MHz, DCM-*d*2, 298 K.

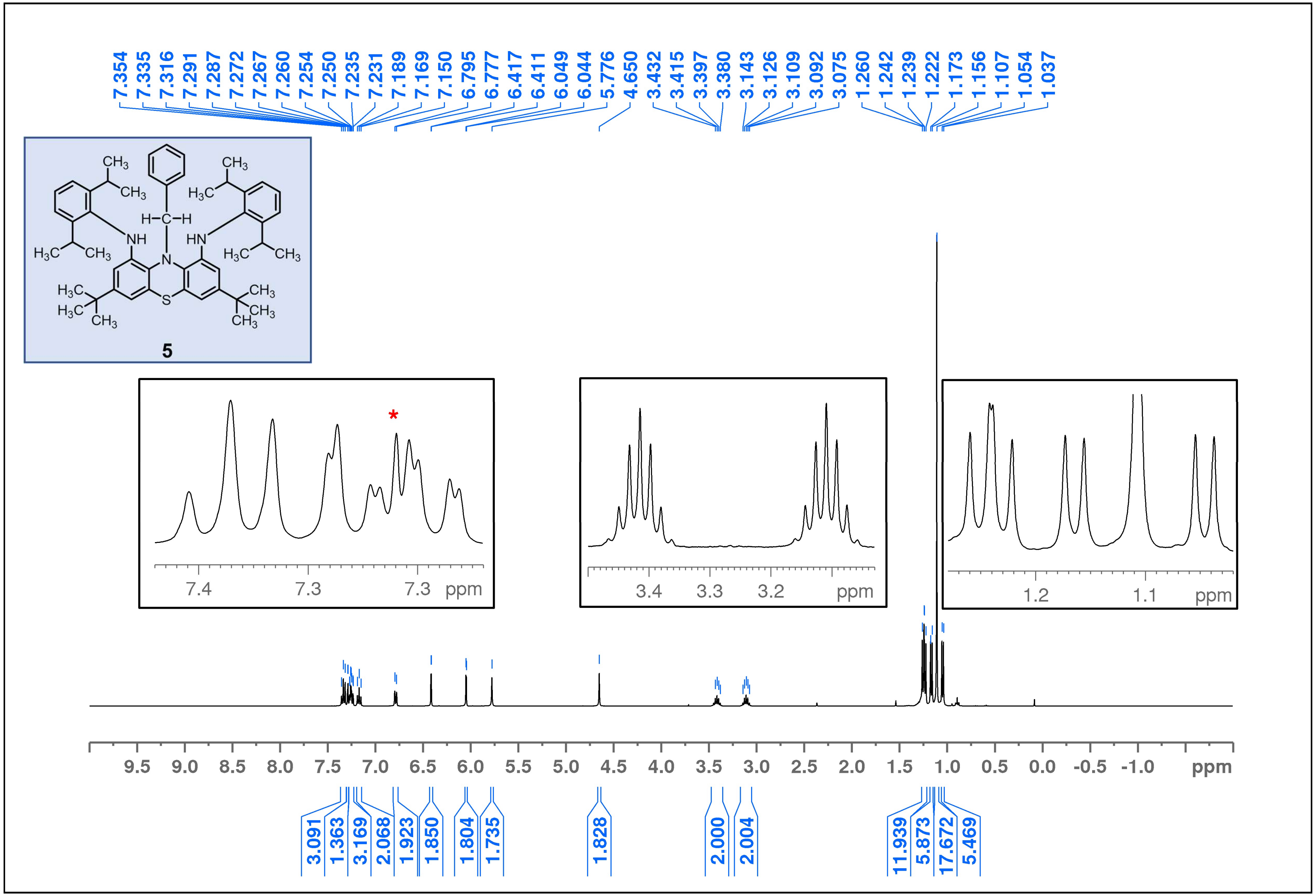

Figure S-6: ^{1}H NMR spectrum of 10-benzyl-3,7-di-*tert*-butyl-N^1,N^9-bis(2,6-diisopropylphenyl)-10*H*-phenothiazine-1,9-diamine (**5**), 400 MHz, chloroform-*d*1, 298 K.

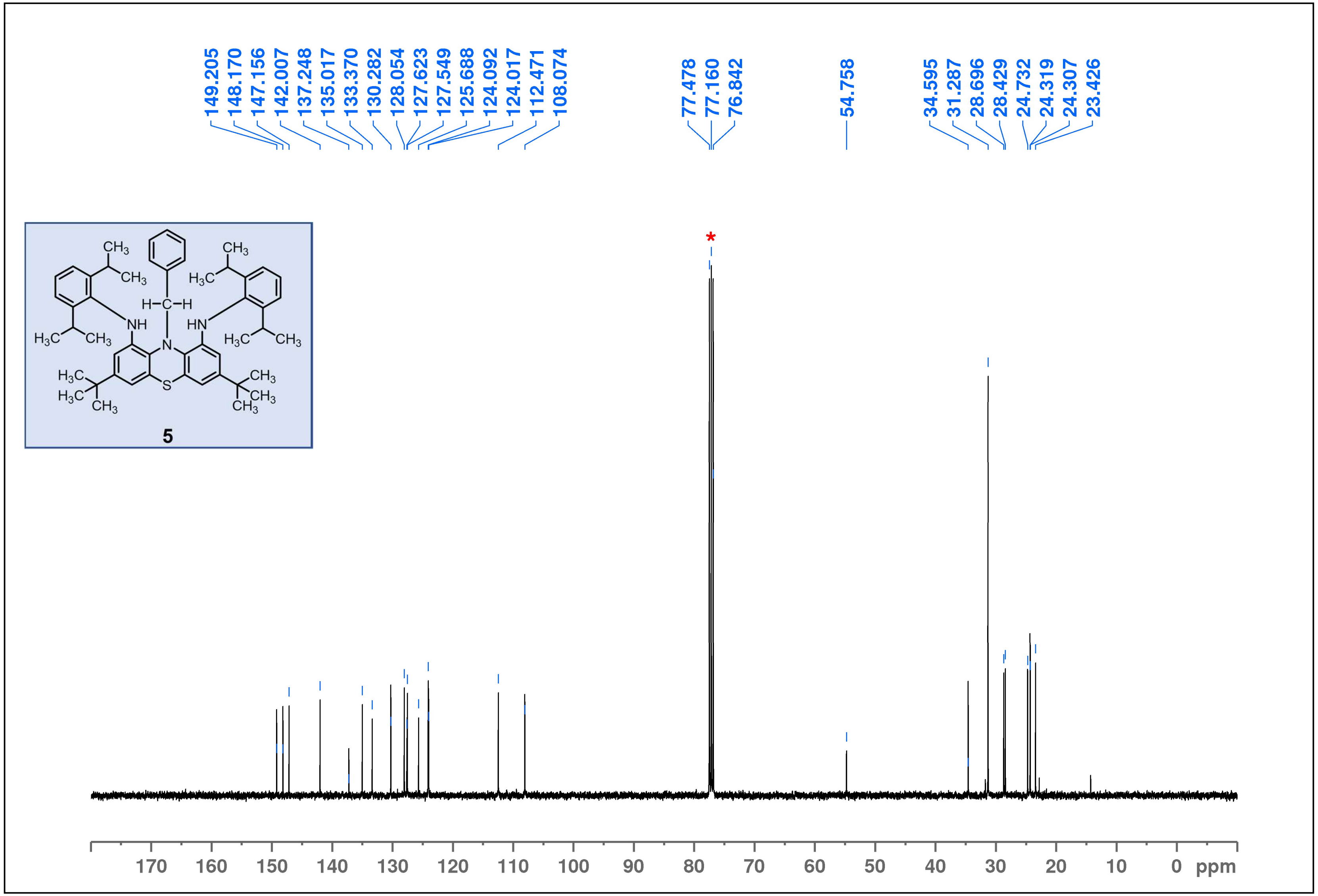

Figure S-7: $^{13}C\{^{1}H\}$ NMR spectrum of 10-benzyl-3,7-di-*tert*-butyl-N^1,N^9-bis(2,6-diisopropylphenyl)-10*H*-phenothiazine-1,9-diamine (**5**), 100 MHz, DCM-*d*2, 298 K.

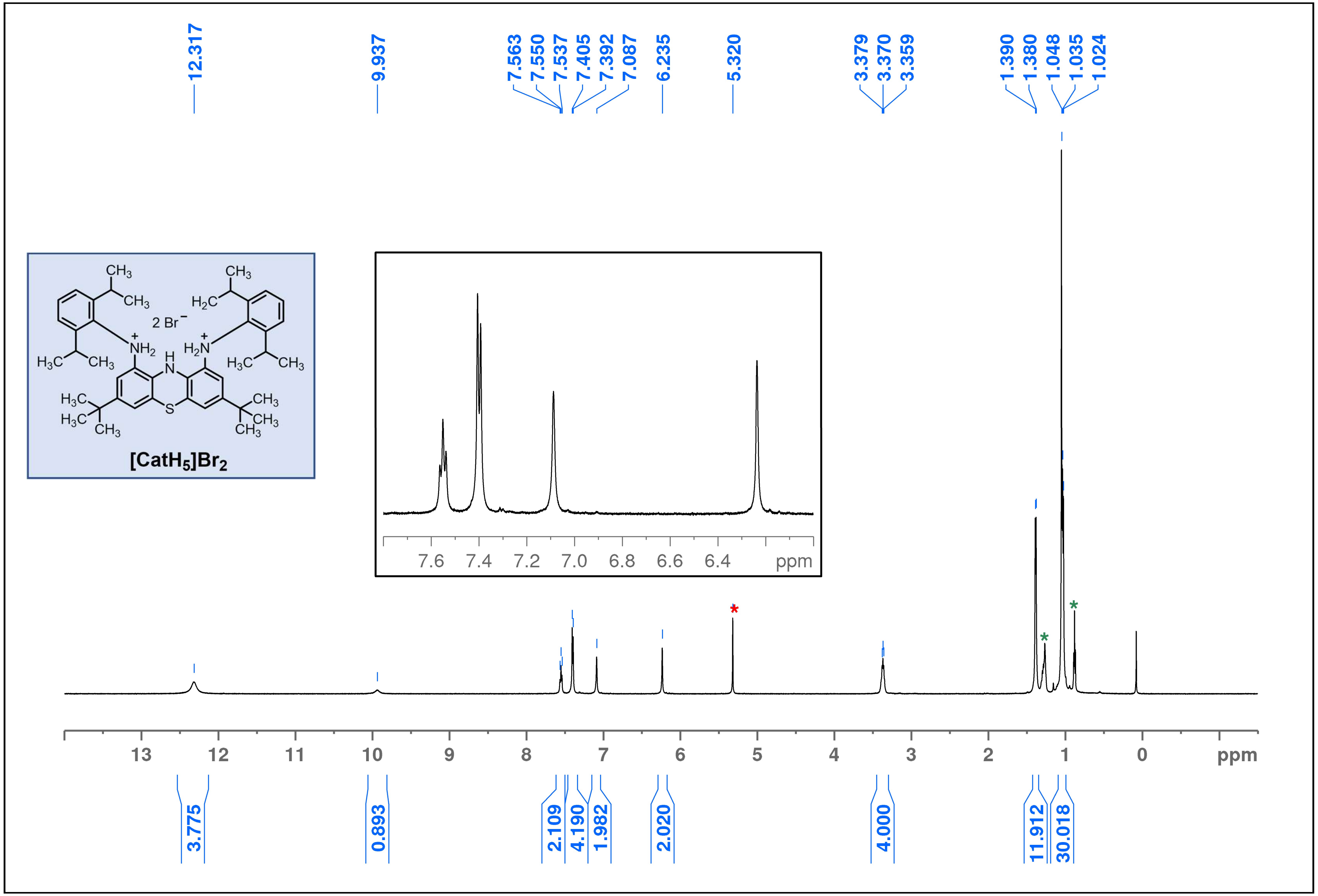

Figure S-8: ^{1}H NMR spectrum of 3,7-di-*tert*-butyl-N^1,N^9-bis(2,6-diisopropylphenyl)-10*H*-phenothiazine-1,9-diaminium bromide ($[\boldsymbol{CatH_5}]\boldsymbol{Br_2}$), 400 MHz, DCM-d2, 298 K, * residual hexane.

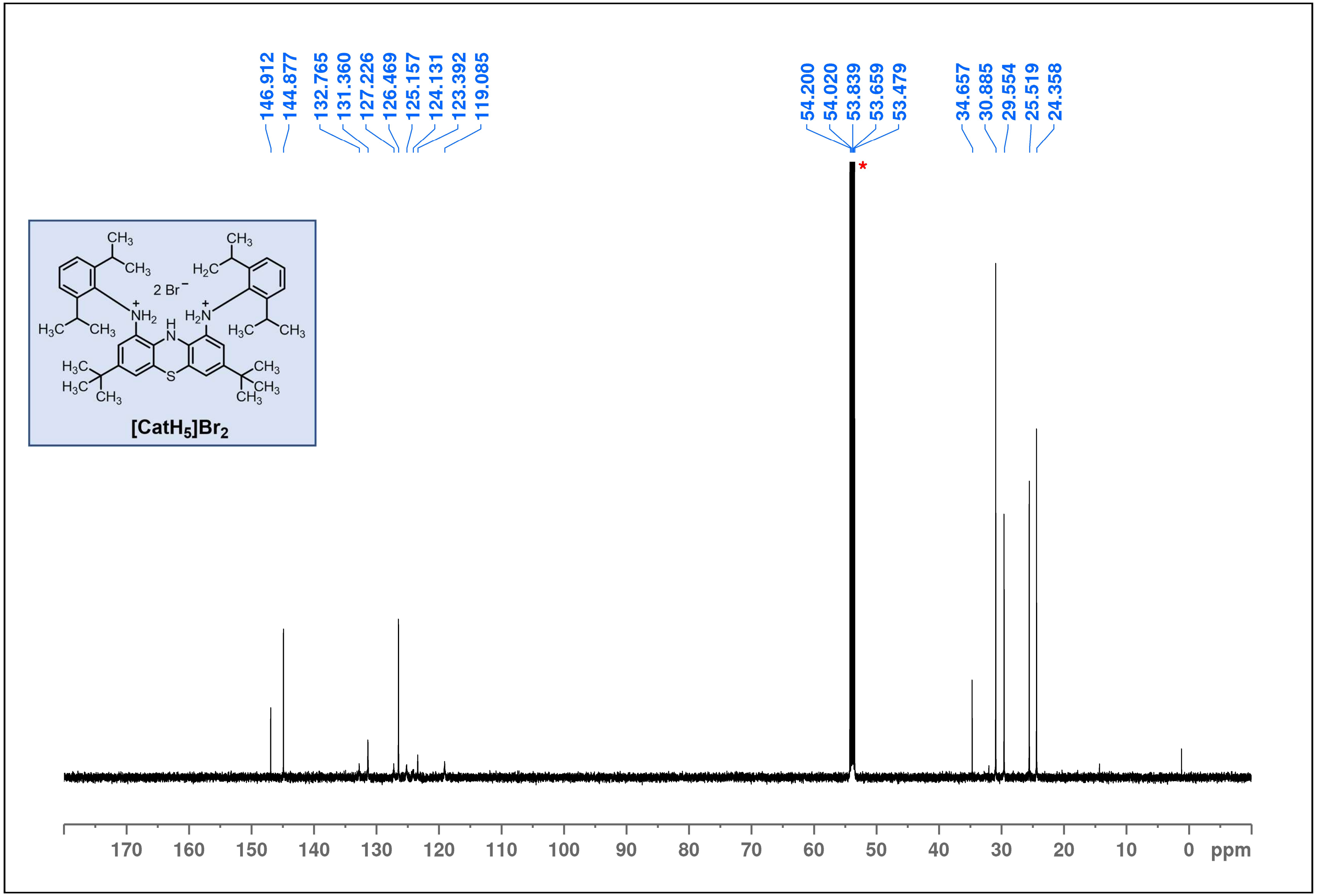

Figure S-9: ^{1}H NMR spectrum of 3,7-di-*tert*-butyl-N^{1},N^{9}-bis(2,6-diisopropylphenyl)-10*H*-phenothiazine-1,9-diaminium bromide ($[\boldsymbol{CatH_5}]\boldsymbol{Br_2}$), 150 MHz, DCM-*d*2, 298 K.

1.4 Evans NMR Study

To follow the synthesis of **$SQH_2^{\bullet}$** and to determine its effectiv magnetic moment, an *in situ* Evans NMR experiment[7] was carried out. For that, the precursor of **$SQH_2^{\bullet}$**, the dihydro dibromide salt **$[CatH_5]Br_2$** (5.0 mg, 6.1 µmol, 1.0 equiv) and cesium carbonate (16 mg, 49.1 µmol, 8.0 equiv) were mixed in DCM-*d*2 in a J. Young NMR tube and degassed water (50 μL) was added under inert conditions. The closed tube was shaken for 3 min and thereafter over a period of 4 h 15 mL of air (3 portions) were led through the reaction mixture. The reaction progress was monitored by ^{1}H NMR spectroscopy. After the reaction was completed, the solvent was removed under reduced pressure and a defined amount of DCM-*d*2 (0.65 mL) was added. The NMR tube was then equipped with a sealed capillary containing pure DCM-*d*2. A subsequent standard ^{1}H NMR measurement (600 MHz, 298.15 K) gave a difference in resonance for the solvent residual signal resulting from the mono-deuterated $CHDCl_2$ of $\Delta\nu = 26.0$ Hz. This difference was used to calculate the molar susceptibility X_M of **$SQH_2^{\bullet}$** (equation (1) and from that the effective magnetic moment μ_{eff} (equation 2).

$$\mathrm{X}_M = \frac{\Delta\nu \cdot M}{\nu_0 \cdot S_f \cdot c} = 1.103 \cdot 10^{-3}\ \mathrm{cm}^3/\mathrm{mol} \quad (1)$$

$$\mu_{eff} = \sqrt{8 \cdot \mathrm{X}_M \cdot T} = \mathbf{1.62}\ \boldsymbol{\mu_B} \quad (2)$$

X_M	molar susceptibility
$\Delta\nu$	difference in resonance for the solvent residual signal ($C\mathbf{H}DCl_2$)
M	molar mass
$\nu_0 = 600.13$ MHz	spectrometer frequency
$S_f = 4\pi/3$	shape factor of the magnet
$c = 6.2\ mg/mL$	concentration
μ_{eff}	effective magnetic moment
T	temperature

1.5 EPR Spectroscopy

Electron paramagnetic resonance spectroscopy of $\mathbf{SQH_2^{\bullet}}$ and $\mathbf{SQH_3^{\bullet+}}$ dissolved in dichloromethane was carried out with a Magenttech Miniscope MS 400 spectrometer at 298 K in the X band frequency range. Simulations of EPR spectra was done with Winsim v.1.0.

Table S-2: Parameters for the simulation of the EPR spectrum of $\boldsymbol{SQH_2^{\bullet}}$.

Compound	Simulation parameter	Value
$SQH_2^{\bullet}$	Domain	CW
	Rel. conc.	100.000
	Line width	0.152
	Lorentzian	24.000
	G-shift	−0.001
	Nuclei	1
	Coupling	0.670
	Spin	1

Table S-3: Parameters for the simulation of the EPR spectrum of $\boldsymbol{SQH_3^{\bullet+}}$.

Compound	Simulation parameter	Value
$SQH_3^{\bullet+}$	Domain	CW
	Rel. conc.	100.000
	Line width	0.270
	Lorentzian	0.000
	G-shift	0.182
	Nuclei	2
	Coupling Nuc. 1	0.670
	Spin Nuc. 1	1
	Coupling Nuc. 2	0.707
	Spin Nuc. 2	0.5

1.6 IR Spectra

Solid state Fourier-transform infrared (FTIR) spectra at 298 K were obtained with an Agilent Cary 630 FTIR spectrometer, which was operated with the MicroLab PC software. Data is reported as follows: absorption wavenumber $\tilde{\nu}$ [cm^{-1}], intensity (*w* = weak, *m* = medium, *s* = strong), band assignment.

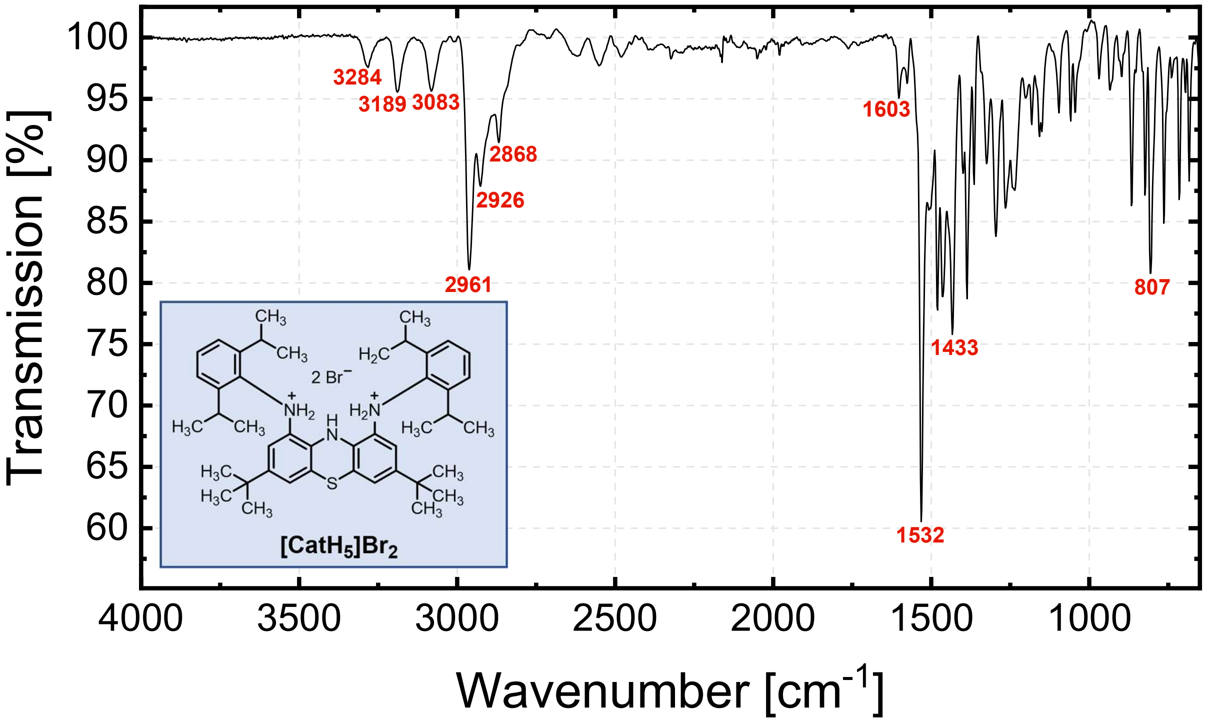

Figure S-10: ATR-FTIR absorption spectrum of 3,7-di-*tert*-butyl-N^1,N^9-bis(2,6-diisopropylphenyl)-10*H*-phenothiazine-1,9-diaminium bromide ([**CatH$_5$**]**Br$_2$**). The spectrum was manually base-line corrected after data acquisition.

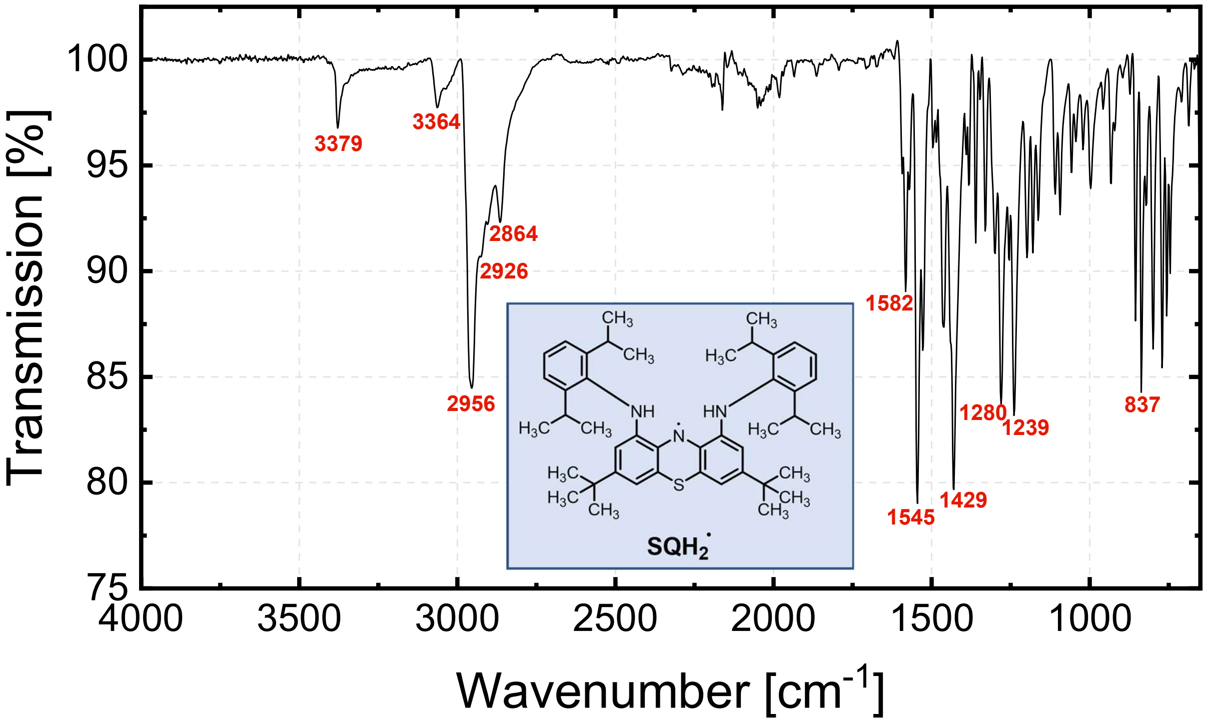

Figure S-11: ATR-IR absorption spectrum of the 1,9-diamino-3,7-di-*tert*-butyl-N^1,N^9-bis(2,6-diisopropylphenyl)-10*H*-phenothiazin-10-yl radical (**SQH$_2^•$**). The spectrum was manually base-line corrected after data acquisition.

1.7 UV-Vis/NIR Spectroscopy

Absorption spectroscopy with near infrared, visible, and ultra violet electromagnetic radiation (UV-Vis/NIR) in solution with dichloromethane as solvent at 298 K was done with a Varian Cary 5000 device using the accompanying Scan software and a standard quartz cuvette ($l = 1$ cm). **[CatH$_5$]Br$_2$** and **SQH$_2^•$** were analyzed with concentrations of approximately 50 µmol/L.

1.8 Mass Spectrometry

Synthesized compounds were characterized with high resolution mass spectrometry (HR-MS) using a direct analysis in real time (DART) ion source or electrospray ionization (ESI) along with a Bruker ApexQe hybrid 9.4 T FT-ICR. Measurements were carried out by the mass spectrometry facility of the organic institute of the University of Heidelberg.

1.9 Cyclic Voltammetry and Spectro-electrochemical Analysis

Cyclic voltammetric (CV) and spectro-electrochemical analyses (SEC) of $\mathbf{SQH_2^{\bullet}}$ was carried out with a Metrohm Autolab PGSTAT 204 potentiostat at 298 K. For UV-Vis absorption measurements the AvaSpec 2048 x 14 CCD spectrometer with a deuterium-halogen light source was employed. The Nova 2.1 and the AvaSoft 8 software were used. The plotted cyclic voltammogram and the map of the SEC analysis shown in the paper in Figure 5 a/c were obtained with the following parameters.

Table S-4: Applied experimental parameters for the CV and SEC analysis of $\boldsymbol{SQH_2^{\bullet}}$.

Experiment	Parameter	Value
**CV**	Solvent	dichloromethane
	Temperature	298 K
	Analyte concentration	0.8 mmol/L
	Supporting electrolyte	tetrabutylammonium hexafluoro-phosphate, 0.25 mol/L
	Reference electrode	Ag/AgCl
	Scan rate	100 mV/s
**SEC**	Solvent	dichloromethane
	Temperature	298 K
	Analyte concentration	0.2 mmol/L
	Supporting electrolyte	tetrabutylammonium hexafluoro-phosphate, 0.25 mol/L
	Reference electrode	Platinum pseudo reference electrode
	scan rate	250 mV/s

1.10 X-Ray diffraction analyses

Suitable crystals of the compounds were taken directly out of the mother liquor, immersed in perfluorinated polyether oil, and fixed on top of a cryo loop. Measurements were made on a Nonius-Kappa charge-coupled device diffractometer with a low-temperature unit using graphite-monochromated Mo-Kα radiation or on a Bruker APEX-III CCD diffractometer diffractometer with a low-temperature unit using Mo-Kα radiation, chromated by mirror optics. The temperature was set at 120 K (Nonius-Kappa diffractometer) or 100 K (Bruker APEX-III CCD diffractometer). The data collected were processed using standard Nonius software[8]or Bruker APEX3 software.[9] Structures were solved by direct methods using the SHELXS or by intrinsic phasing using the SHELXT

program and refined with the SHELXL.[10] Graphical handling of the structural data during solution and refinement was performed with Olex2 v1.3.[11] Atomic coordinates and anisotropic thermal parameters of non-hydrogen atoms were refined by full-matrix least-squares calculations [10c, 12] and displacement ellipsoides are displayed with 50 % probability. Hydrogen atoms were included using a riding model.

Crystallographic data for the structure reported in this article are deposited with the Cambridge Crystallographic Data Centre as supplementary publication no. CCDC 1965582 (**[CatH$_5$]Br$_2$**), no. CCDC 1965576 (**SQH$_2$**), no. CCDC 1965577 (**[QH$_2$]Br**) and can be obtained free of charge. Crystal data and structure are summarized in the tables under the crystallographic figures.

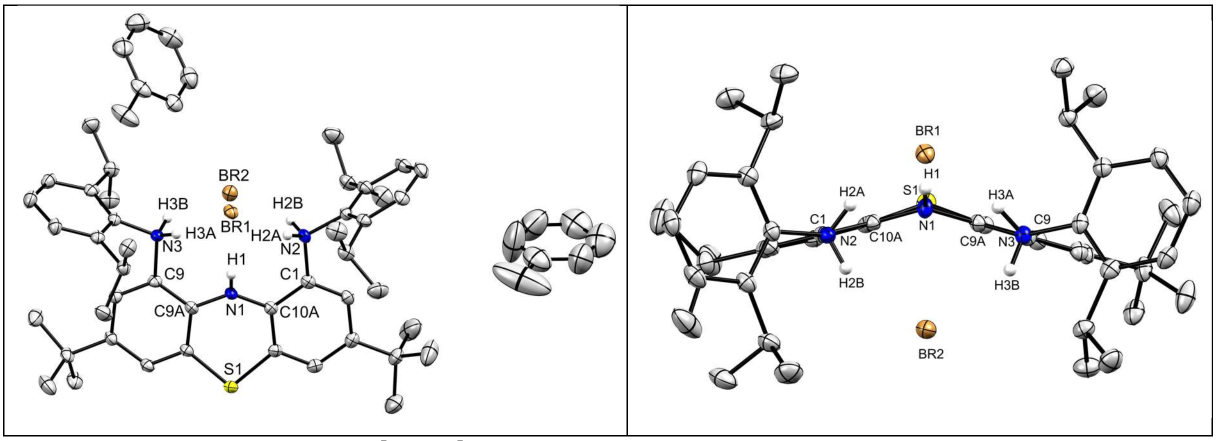

Figure S-12: Molecular structure of $[CatH_5]Br_2$. Non-relevant hydrogen atoms are omitted for clarity. Displacement ellipsoids are drawn with a probability of 50 %. Left: top view; Right: front view, toluene molecules are omitted for clarity. Selected bond distances [pm]: N(2)-C(1) 148.4(4), C(1)-C(10A) 140.3(4), C(10A)-N(1) 139.3(4), N(1)-C(9A) 139.3(3), C(9A)-C(9) 139.3(4), C(9)-N(3) 148.6(3).

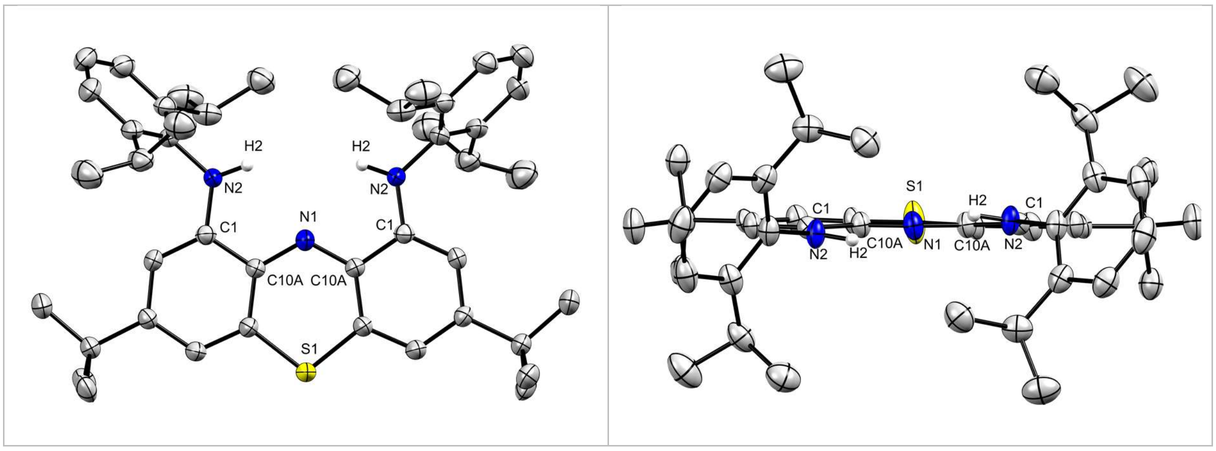

Figure S-13: Molecular structure of $SQH_2^{\bullet}$. Non-relevant hydrogen atoms are omitted for clarity. Displacement ellipsoids are drawn with a probability of 50 %. Left: top view; Right: front view. Selected bond distances [pm]: N(2)-C(1) 137.7(2), C(1)-C(10A) 142.7(3), C(10A)-N(1) 135.9(2).

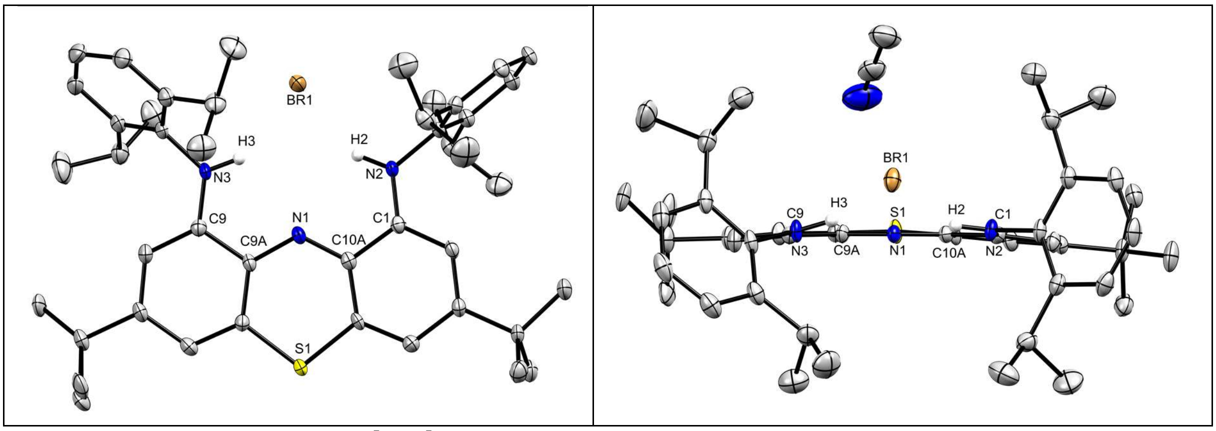

Figure S-14: Molecular structure of $[\mathbf{QH_2}]\mathbf{Br}$. Non-relevant hydrogen atoms are omitted for clarity. Displacement ellipsoids are drawn with a probability of 50 %. Disorder at ᵗButyl group is omitted for clarity in both pictures. Left: top view, acetonitrile molecule is omitted for clarity; Right: front view. Selected bond distances [pm]: N(2)-C(1) 133.4(2), C(1)-C(10A) 145.3(3), C(10A)-N(1) 132.5(2), N(1)-C(9A) 133.7(2), C(9A)-C(9) 145.2(3), C(9)-N(3) 134.7(2).

Comparison of bond lengths obtained by computation and experiment

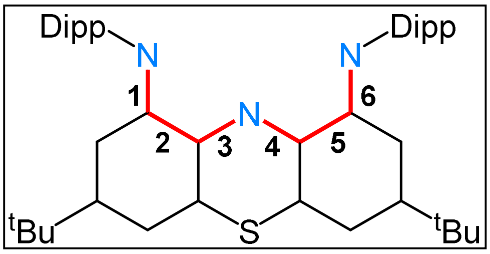

Table S-5: Selected Bond lengths obtained by SCXRD in comparison with calculated values for $[\boldsymbol{CatH_5}]\boldsymbol{Br_2}$, $\boldsymbol{SQH_2^{\bullet}}$, and $[\boldsymbol{QH_2}]\boldsymbol{Br}$. Calculations were carried out on the TPSS-D3(BJ)/def2-TZVPP and for $\boldsymbol{SQH_2^{\bullet}}$ on the UTPSS-D3(BJ)/def2-TZVPP level of theory. Values are given in pm.

Bond number	X-ray $[CatH_5]Br_2$	Calc. $[CatH_5]Br_2$	X-ray $SQH_2^{\bullet}$	Calc. $SQH_2^{\bullet}$	X-ray $[QH_2]Br$	Calc. $[QH_2]Br$
**1**	148.6(3)	148.6	137.7(2)	137.9	134.7(2)	133.8
**2**	139.3(4)	139.8	143.0(3)	143.6	145.2(3)	145.8
**3**	139.3(3)	139.5	136.4(2)	135.7	133.7(2)	133.4
**4**	139.3(4)	139.5	136.4(2)	135.7	132.5(2)	133.2
**5**	140.3(4)	140.1	143.0(3)	143.6	145.3(3)	145.7
**6**	148.4(4)	148.4	137.7(2)	137.9	133.4(2)	133.6

Table S-6: Details of the X-ray crystal structure analysis of $[\boldsymbol{CatH_5}]\boldsymbol{Br_2}$, $\boldsymbol{SQH_2^{\bullet}}$, and $[\boldsymbol{QH_2}]\boldsymbol{Br}$.

Identification code	$[CatH_5]Br_2$	SQH_2	$[QH_2]Br$
Empirical formula	$C_{58}H_{77}Br_2N_3S$	$C_{44}H_{58}N_3S$	$C_{46}H_{61}BrN_4S$
Formula weight	1008.1	661.00	781.95
Temperature/K	100	120	100
Crystal system	orthorhombic	monoclinic	monoclinic
Space group	$P2_12_12_1$	C2/c	$P2_1/n$
a/Å	12.1245(6)	14.704(3)	10.4106(19)
b/Å	15.7003(8)	19.701(4)	14.956(3)
c/Å	28.6225(14)	14.110(3)	27.730(5)
α/°	90	90	90
β/°	90	106.07(3)	97.099(6)
γ/°	90	90	90
Volume/Å^3	5448.5(5)	3927.8(15)	4284.6(13)
Z	4	4	4
ρ_{calc} g/cm^3	1.229	1.119	1.212
μ/mm^{-1}	1.564	0.115	1.043
F(000)	2128	1440.0	1664
Crystal size/mm^3	0.246 × 0.083 × 0.075	0.55 × 0.45 × 0.35	0.332 × 0.110 × 0.109
Radiation	MoKα (λ = 0.71073)	MoKα (λ = 0.71073)	MoKα (λ = 0.71073)
2Θ range for data collection/°	3.85 to 57	4.1 to 56.986	4.022 to 57
Index ranges	-16 ≤ h ≤ 16, -21 ≤ k ≤ 21, -38 ≤ l ≤ 38	-19 ≤ h ≤ 19, -26 ≤ k ≤ 26, -18 ≤ l ≤ 18	-13 ≤ h ≤ 13, -20 ≤ k ≤ 20, -37 ≤ l ≤ 36
Reflections collected	307892	9718	107505
Independent reflections	13814 [R_{int} = 0.0772, R_{sigma} = 0.0273]	4983 [R_{int} = 0.0674, R_{sigma} = 0.0932]	10837 [R_{int} = 0.0831, R_{sigma} = 0.0435]
Data/restraints/parameters	13814/80/593	4983/0/225	10837/0/515
Goodness-of-fit on F^2	1.037	1.000	1.045
Final R indexes [I≥2σ (I)]	R_1 = 0.0284, wR_2 = 0.0708	R_1 = 0.0591, wR_2 = 0.1394	R_1 = 0.0426, wR_2 = 0.0950
Final R indexes [all data]	R_1 = 0.0325, wR_2 = 0.0731	R_1 = 0.1418, wR_2 = 0.1729	R_1 = 0.0585, wR_2 = 0.1025
Largest diff. peak/hole /e Å^{-3}	0.49/-0.47	0.43/-0.52	0.56/-0.38
Flack parameter	0.0065(19)	-	-
CCDC deposition number	1965582	1965576	1965577

2 Computational Details

All quantum chemical calculations were performed with Orca 4.0.1[13] using the computational resources of the bwUniCluster at the Karlsruhe Institute of Technology (KIT) within the Baden-Württemberg High Performance Computing program (bwHPC).

For all density functional theory calculations the chain of spheres exchange approximation (COSX)[14] combined with the Split-RI-J algorithm[15] (RIJCOSX) was used. Also, the D3 version of Grimme's empirical dispersion correction[16] along with Becke-Johnson damping[17] was always included. All molecular structures were optimized using the meta-GGA TPSS functional[18] with the def2-TZVPP basis set[19] as implemented in Orca 4.0.1. Radical stabilization energies (*vide infra*) were obtained with B3LYP[20] as functional and the 6-31G(d)[21] basis set. Results from geometry optimizations were verified by computing the Hessian matrix on the same level of theory. Equilibrium geometries were confirmed to possess no negative Hessian matrix eigenvalue. For the calculation of vertical electronic excitations, the combination of B3LYP with def2-TZVPP was shown most accurate, when comparing calculated data with experimentally obtained spectra. The first 60 excited states were included in the simulation of UV-Vis/NIR absorption spectra, which were obtained by fitting the calculated vertical electronic excitations with Lorentz peak functions. NBO spin density analysis of the radicals was performed on the TPSS/def2-TZVPP derived spin density distribution with the NBO 7.0 program. (NBO 7.0. E. D. Glendening, J. K. Badenhoop, A. E. Reed, J. E. Carpenter, J. A. Bohmann, C. M. Morales, P. Karafiloglou, C. R. Landis, and F. Weinhold, Theoretical Chemistry Institute, University of Wisconsin, Madison, WI (2018))

2.1 Radical Stabilization Energy

Radical stabilization energies (RSE) were computed following the scheme from HIOE *et al.*[22]

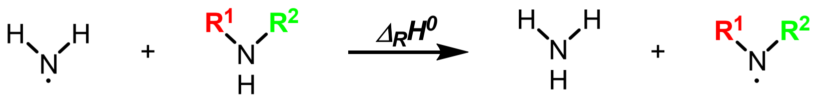

$$\Delta_R H^0 = H^0(NH_3) + H^0(\cdot NR^1R^2) - H^0(\cdot NH_2) - H^0(HNR^1R^2) \tag{3}$$

$$\Delta_R H^0 = H^0(NH_3) + H^0(\cdot NR^1R^2) - H^0(\cdot NH_2) - H^0(HNR^1R^2) + H^0(\cdot H) - H^0(\cdot H) \tag{4}$$

$$\Delta_R H^0 = H^0(\cdot H) + H^0(\cdot NR^1R^2) - H^0(HNR^1R^2) - (H^0(\cdot H) + (\cdot NH_2) - H^0(NH_3)) \tag{5}$$

$$\Delta_R H^0 = \boldsymbol{RSE}(\cdot \boldsymbol{NR^1R^2}) = BDE(HNR^1R^2) - BDE(NH_3) \tag{6}$$

The standard reaction enthalpy of the reference reaction shown above represents the difference in N-H bond dissociation energy and therefore is interpreted as the radical stabilization energy.

2.2 xyz Coordinates

Illustrations of equilibrium structures, molecular orbitals, or spin densities were obtained with IboView v20180510.[23]

10*H*-Phenothiazine (**1**)

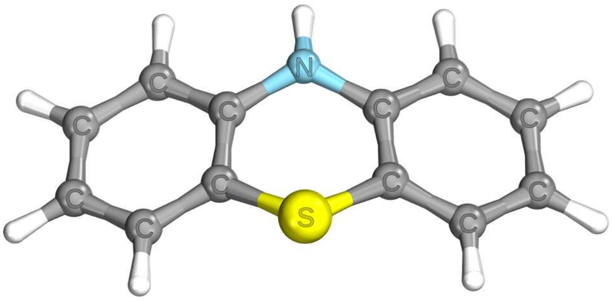

B3LYP-D3(BJ)/6-31G(d)

xyz 0 1

C	-6.48091654451965	2.29903265748510	-0.33301162044622
C	-6.77470837262837	0.94050523926449	-0.45035259703364
C	-5.80942375592683	-0.00501187856626	-0.09757059667865
C	-4.56957823672157	0.39936758661709	0.39761027323436
C	-4.26635354277865	1.76812223354682	0.50477767058010
C	-5.22900493097311	2.71002755665986	0.12448193269330
N	-3.01613752915018	2.17963833991560	0.99431605734122
C	-1.84312312952312	1.45252589196349	0.73596440164825

C	-1.88398776581272	0.04972642273271	0.65052535513842
S	-3.40620429740487	-0.81354385277921	0.99277853265811
H	-4.99302469944901	3.76985929935347	0.19573688168615
H	-7.21950263146886	3.04563466443396	-0.61037488129101
H	-7.74379318936573	0.61490241341232	-0.81589261913795
H	-6.01997503340049	-1.06668027612084	-0.19108025069791
C	-0.61899068876114	2.10946643196825	0.56779512982862
C	0.54930827187666	1.38318721974409	0.33751134526393
C	0.50362424862474	-0.00726363154677	0.23722313875873
C	-0.71859742606525	-0.66767276992828	0.38011769785801
H	1.40798200375438	-0.57677597445852	0.04572218912840
H	-0.77155388298570	-1.74959125392619	0.29757968852059
H	-0.58629276352686	3.19563167337544	0.62431558133126
H	1.49189329931887	1.91065165304706	0.22222741133948
H	-2.88481940311253	3.18330035380632	0.98970927827647

Phenothiazin-10-yl radical (**1-SQ**$^{•}$)

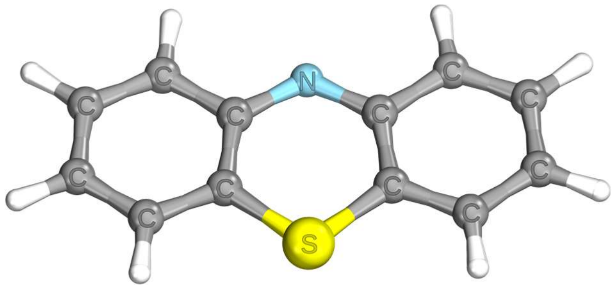

UB3LYP-D3(BJ)/6-31G(d)

xyz 0 2

C	-6.57292267408266	2.31727439819711	-0.02299415956258
C	-6.89258694290375	0.95337789754409	-0.13923878445464
C	-5.88350606217345	-0.00290659848166	-0.09779302657381
C	-4.54772456003858	0.38363553181581	0.05905787063548
C	-4.19535086664812	1.76177585637495	0.18070677234306
C	-5.25674994878357	2.70753066543962	0.13294901617045
N	-2.94293332277731	2.26458819542944	0.33885569023011
C	-1.85249750468031	1.45643242755124	0.40263149757264
C	-1.84564328546854	0.03154538898980	0.31434089805033
S	-3.33090306648524	-0.88608131198693	0.09784744250997
H	-4.97729545628297	3.75210227005895	0.22523813346397
H	-7.36088028075358	3.06420193568759	-0.05583338106870
H	-7.92529499639320	0.64038007169747	-0.26119140900976
H	-6.12799772744253	-1.05844124224593	-0.18696219876280
C	-0.59581205283593	2.09992016644968	0.57584661820259
C	0.58333690158142	1.38436917507332	0.65617298485281
C	0.56192431651674	-0.01830104257785	0.56695065970273
C	-0.64661334999157	-0.68536233220860	0.39705546371016
H	1.48524062455185	-0.58644280776719	0.63091521120411
H	-0.66601007384471	-1.77018868270178	0.32852481185577
H	-0.61313617295381	3.18302396726564	0.64175825466439
H	1.52701650188980	1.90561607039524	0.78921163426371

3,7-Dimethoxy-10*H*-phenothiazine

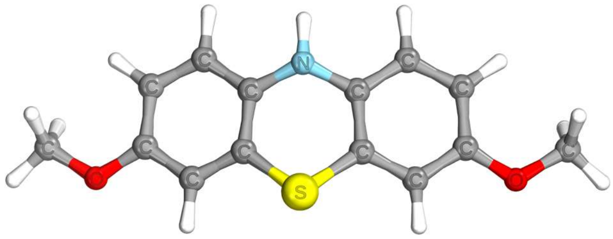

B3LYP-D3(BJ)/6-31G(d)

xyz 0 1

C	-6.51455249924047	2.21396491725354	-0.20150727040285
C	-6.72913320586879	0.83685428719454	-0.29371210542352
C	-5.70481091271533	-0.04706044248532	0.07367381256318
C	-4.49576442299430	0.43700977631629	0.55843149019809
C	-4.26481424311068	1.82323151584565	0.64820645476103
C	-5.28007454847609	2.69209119032508	0.24992790255754
N	-3.04131831050458	2.30623464132276	1.14915944580632
C	-1.83065447790421	1.64169700152861	0.87833574035286
C	-1.79360117173176	0.23559584170351	0.81140716776142
S	-3.26684857191324	-0.70620463917226	1.15890778151261
H	-5.11249696618360	3.76593908457921	0.30072338646847
H	-7.28204900468797	2.92353411134626	-0.48618139436675
H	-5.87943429391169	-1.11454211917099	-0.01329256363464
C	-0.64229585291770	2.34630205041885	0.69035331065857
C	0.56996414197462	1.68591988344808	0.46614816677186
C	0.59270296237282	0.29137429611792	0.39020679862688
C	-0.60063643820548	-0.42729142438866	0.54909865785311
H	-0.57265772840304	-1.50975593513060	0.47811664785788
H	-0.65654059546838	3.43360857948078	0.72761801913162
H	1.47280678844264	2.27112046632071	0.33992187189455
H	-2.96380473886240	3.31514870638181	1.12526980264809
O	-7.88618438280006	0.25164942775965	-0.72958973617687
C	-8.94657764879693	1.10543336141425	-1.11696681505464
H	-9.28418288881232	1.73537562874861	-0.28195356810207
H	-9.76294943265578	0.45065418292413	-1.42830065716751
H	-8.65834951149488	1.75166067755067	-1.95798562741824
O	1.71141156349440	-0.46224891772527	0.16141615179504
C	2.93747509181920	0.22255716929169	-0.01765417435175
H	2.90240033087694	0.89328714484239	-0.88758715975667
H	3.69176834620639	-0.54831170143174	-0.18718414976430
H	3.20816295647266	0.80647748038987	0.87322004440069

3,7-Dimethoxyphenothiazin-10-yl radical

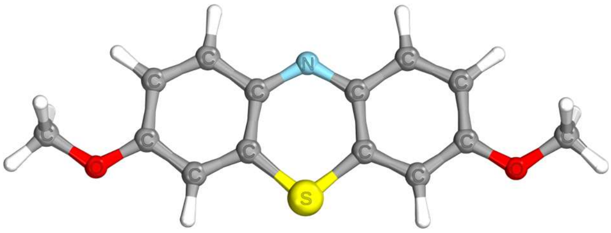

UB3LYP-D3(BJ)/6-31G(d)

xyz 0 2

C	-6.59330434343577	2.21595185166063	-0.07698035836448
C	-6.83315505787224	0.83459923020660	-0.19355861238076
C	-5.75956295198116	-0.06352577524184	-0.15619917169621
C	-4.45679785700249	0.39843318929748	-0.00614157963623
C	-4.17716716376256	1.79511775625689	0.11532066768411
C	-5.29357965780397	2.66880232757051	0.07273041491541
N	-2.95461937953474	2.36853622078878	0.26783416176726
C	-1.82165893641986	1.62017694904272	0.32700418155713
C	-1.73202621791926	0.19613901064459	0.23849465577303
S	-3.16801955458461	-0.80630063531158	0.02367032728219
H	-5.08165887964363	3.72929471837597	0.16387768157636
H	-7.40926626243901	2.92834568990282	-0.10333552130437
H	-5.97149952068604	-1.12469048371744	-0.24774945340789
C	-0.60001020328637	2.32033413999160	0.49579834712954
C	0.62518032910206	1.68023834975732	0.57433682978722
C	0.67737619006481	0.27712480033241	0.48551105657246
C	-0.50528612637598	-0.45336211607684	0.31716016556181
H	-0.43820869570745	-1.53529386450611	0.25185535455352
H	-0.66779324723885	3.40149042253271	0.56232476477420
H	1.52769484679944	2.26556424920874	0.70379926445462
O	-8.06388946604232	0.27308799203218	-0.34555049703592
C	-9.18953734021900	1.13490330661290	-0.41002758892853
H	-9.30530854663265	1.71484110505895	0.51508629112521
H	-10.05632436212654	0.48471637015365	-0.54075399246334
H	-9.11896513454838	1.82357266784702	-1.26223106884049
O	1.81966201833283	-0.46088169744354	0.55163713590523
C	3.04908725311630	0.22451844012881	0.73248021000344
H	3.25243952742102	0.91133738860033	-0.09964335166311
H	3.82011570866245	-0.54734414901758	0.76151156948428
H	3.06020303176398	0.78636254531130	1.67580811581432

3,7-Di-*tert*-butyl-N^1,N^9-bis(2,6-diisopropylphenyl)-10*H*-phenothiazine-1,9-diamine (**CatH$_3$**)

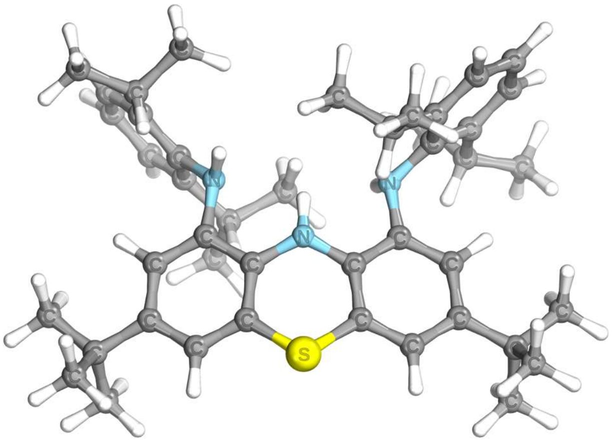

B3LYP-D3(BJ)/6-31G(d)

xyz 0 1

C	-4.62883082405255	-0.96614056509095	-4.49969335957875
C	-4.77968450458517	-3.39261385482159	-3.82636318228765
C	4.84441050001135	1.19275228770260	-4.29034577124729
C	-4.66195918496099	-1.94426495922377	-3.30424537336498
C	5.94792193025306	-0.84841687561859	-3.29861036263166
C	-2.12649437139888	-2.03522927942306	-3.11000253707719
C	-5.91058714827343	-1.62948571825562	-2.46261197904254
C	2.89876004733907	-0.76266550854262	-2.96348231046964
C	-3.36670422313560	-1.78814869608466	-2.49622829650365
C	5.14691615365316	0.43327759177161	-2.98010759274106
C	-0.92907807959331	-1.87819682105656	-2.41609332323371
C	1.71254021433902	-1.16845665077340	-2.35613661247200
C	3.82852898882025	0.05090435263610	-2.29290269481365
C	6.02122670563424	1.33656818453053	-2.09397856711462
C	-3.36195422886982	-1.38609607962756	-1.15797472521943
C	-0.93607266269646	-1.41994086647489	-1.09101678177997
C	-3.07586247013718	2.96787832318191	-1.13885752791422
C	1.39930553431689	-0.73436633303566	-1.05950432607912
C	3.49166491836505	0.49473543029725	-1.01111259708307
C	-2.16716096350692	-1.18980479040100	-0.45988133009339
C	-2.44969479531860	1.93369963790744	-0.19480541529577
C	2.28097033614484	0.14221751045512	-0.40985074479544
C	-1.22577906006361	2.51933381835014	0.53496597047865
C	-3.42317732946880	1.36080558265476	0.82950982728144
C	-3.21806280961781	0.07128356396277	1.37210228540421
C	4.17680199271472	4.27827471342527	1.21585486327686
C	-4.48801055106487	2.12048351202977	1.31928031171708
C	3.03949055079654	3.27133371802029	1.43990761533485
C	2.89302271133898	0.76329728231409	1.88272375635417
C	3.38107202946068	-2.97007127708533	2.64121650217708
C	3.47668485450561	2.01228470882935	2.17882225051129
C	2.56024685252972	-1.72419497909119	2.28559621646200
C	1.85757659520451	3.94129742469934	2.16982259349746
C	-4.05325271116025	-0.43759346799698	2.38938993609154
C	3.27262590206560	-0.40866325808928	2.57360372329462

C	-5.31378824907130	1.63894615613122	2.33445634300962
C	-3.80049518725857	-1.82372013810463	2.96945055036425
C	-5.08813771650057	0.37420751387333	2.86874483929002
C	4.43688017732781	2.07229604439226	3.19734172497189
C	1.19840814984506	-1.75018843842395	3.00982918155376
C	4.25253166534432	-0.30495746796616	3.56321529450575
C	-5.06119385820343	-2.50515306043484	3.51981392533239
C	-2.70671205141574	-1.76494669783044	4.05703844213997
C	4.82958352535537	0.92533547000997	3.87943024519500
H	-5.54632387596649	-1.05537998088093	-5.09466235757776
H	-3.77896358044355	-1.16422212444337	-5.16125880657290
H	-4.54584402357070	0.06930065398537	-4.14985224107161
H	-5.69826531591402	-3.51466746197845	-4.41365867744020
H	4.25864601233612	0.58416378709377	-4.98716955752383
H	-3.93426511417055	-3.66227708417334	-4.46800044493943
H	5.77780448172952	1.47381222510780	-4.79358839693072
H	-2.08387673261642	-2.35409698645394	-4.14674777390819
H	5.38846530051139	-1.52175036344224	-3.95650221438136
H	6.89150593590444	-0.59646905117611	-3.79830986754922
H	3.10391727227579	-1.10646460563181	-3.97288176338929
H	-6.80883114206817	-1.74268627669282	-3.08004368027924
H	4.27659140125452	2.10743287196368	-4.08510014183667
H	-4.80844963158896	-4.10417988830606	-2.99320233678096
H	-5.89480658269892	-0.60166294026689	-2.08208813944163
H	6.18215999734247	-1.39651401606513	-2.37880083157310
H	6.95288647616882	1.57596845138420	-2.61877933421072
H	-6.00600796145473	-2.30926477866315	-1.60807772109221
H	-2.35650202597741	3.24314038410467	-1.91855057239067
H	-3.97034384044021	2.56584948978966	-1.62792191232742
H	5.51893359591654	2.28240947363551	-1.85931018176409
H	-4.28704209455006	-1.18323689703340	-0.63336381186786
H	6.28673530279146	0.84542180087092	-1.15110538884182
H	-2.09036347150141	1.11700638901775	-0.82455090388167
H	-3.35798482874357	3.89031669254474	-0.61752163260427
H	-0.46780281884901	2.85482437579356	-0.18564094020602
H	4.16272061277996	1.13185104300433	-0.44865029826859
H	0.10409837416206	-0.66591880927989	0.47989611134096
H	2.69415414838002	2.97097813224077	0.44221657963708
H	-0.77965328517484	1.76702924096414	1.19284328070481
H	-1.51281345271588	3.38043423702301	1.15040259651105
H	3.83132888072283	5.09524836614898	0.57197776076678
H	5.03976170046025	3.80329930379997	0.73582937662063
H	-1.81651512649440	-1.41015471640701	1.53750620826052
H	1.35145729511212	1.50450176872202	0.77358575155557
H	-4.66855483040431	3.11121177515235	0.91502101810656
H	2.35949686070701	-1.77367088846236	1.21215862395779
H	4.36836339125020	-2.94757176710779	2.16652321635151
H	1.49262427457775	4.81269779816171	1.61263974344286
H	2.85817570084856	-3.86906341069806	2.29579940842654
H	-3.43476936261025	-2.46099686166806	2.15191272355484
H	4.51674817452936	4.72687313003280	2.15629587100614
H	1.01970122516514	3.24866201467673	2.30594962751703
H	-5.86609498549132	-2.51300784224422	2.77686560272986
H	-6.13083794582693	2.25042144430822	2.70760652650006
H	3.52560691835411	-3.07330368265802	3.72347307809630

H	0.62959137114123	-2.64617121404518	2.73068478267355
H	-1.77764649359817	-1.31375368551772	3.68937637304661
H	2.16952973424360	4.27683775892543	3.16603228650074
H	4.89467479815869	3.02427676511744	3.44774569960494
H	0.60455825859865	-0.86606959258963	2.75656431272033
H	-4.83608334443741	-3.54214544845784	3.79316998141238
H	-5.73853697378589	0.00475686601706	3.65491657023740
H	4.56148170868579	-1.19190748984254	4.10644969950523
H	-2.47104904248259	-2.76803855639027	4.43223668178656
H	1.34036222335337	-1.76089538864395	4.09740813086893
H	5.58467215791290	0.98772445650729	4.65857431663107
H	-5.43400341859908	-2.00536364014471	4.42139042706787
H	-3.04870722818324	-1.15435472829938	4.90108538663980
N	0.24627655378242	-1.16091285655276	-0.39621759952999
N	-2.12642424990006	-0.70115243056569	0.88271532816687
N	1.87952369955838	0.64464837225985	0.87991949793873
S	0.62488158653108	-2.32109064729343	-3.18333603911157

3,7-di-*tert*-butyl-*N*[1],*N*[9]-bis(2,6-diisopropylphenyl)-10*H*-phenothiazine-1,9-diaminium bromide ([**CatH$_5$**]**Br$_2$**)

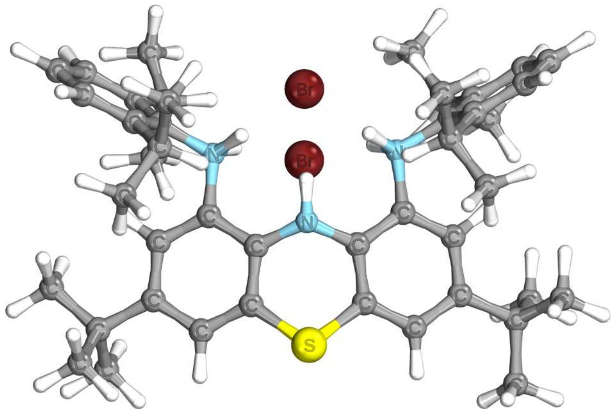

TPSS-D3(BJ)/def2-TZVPP

xyz 0 1

Br	3.13308655193186	2.72297297183006	20.05984710786515
Br	5.01199655072639	2.25232143520117	16.04604009669561
S	8.03767883703422	6.18392738006856	18.65700679230724
N	5.74006909438917	4.33248657831619	18.85896559590750
H	4.91782582181248	3.79945467410318	19.16636030761340
N	3.24234055439149	4.54112970291002	17.38689567083212
H	3.20666788232362	4.08578924132115	18.34449007958112
H	3.56135247310731	3.72806675383214	16.77338180966287
N	5.89148685732243	1.43084899025883	19.02086808851166
H	5.05411288942654	1.90328736423227	19.47565708750105
H	5.58558355061304	1.38484213540173	18.00220519026287
C	4.31059792387615	5.56586433056028	17.28048436042605
C	6.34902687101846	7.38487667175975	16.84603220024092
H	7.17470924477722	8.06975714090846	16.69023629973749
C	5.51635788424238	5.38512737345849	17.97089649346224

C	6.53116700170956	6.32368659296083	17.73278684698147
C	4.11649039945264	6.64156763096016	16.42760802490759
H	3.15788779444773	6.73696967381192	15.93107877642598
C	7.10255269089727	2.29111520480949	19.04175729418834
C	6.97017946635188	3.67479850336814	18.89162585567129
C	6.05334711567447	0.10794550541520	19.66523578158411
C	5.12821132464841	7.57893793493217	16.19380593416431
C	9.52694714532255	2.42261233882283	19.02519982377139
C	6.44574844861956	-2.31994911166726	20.91035417242980
H	6.60464594631955	-3.27723415910814	21.39798943331645
C	6.05760933431486	-1.04997169640389	18.87054127484298
C	6.41012400131611	-1.15324921490772	21.66429897836328
H	6.53411250805919	-1.20200117634667	22.74165970738953
C	6.20693004735437	0.09179020746859	21.06016518556357
C	9.40383990075408	3.80339801399108	18.81407889440084
H	10.28867358397484	4.42290419320238	18.70465018733578
C	8.15828134522173	4.41850478387103	18.77238674206655
C	1.88032383137856	5.03479953470382	17.08741212314528
C	4.86216991895539	8.74817773704041	15.24232109717736
C	1.29376756746739	4.72240781261163	15.85264482202476
C	6.26091953344754	-2.26640680690933	19.53239864386169
H	6.26806233821358	-3.18468231242023	18.95616858970872
C	8.35064158764598	1.68216239962274	19.13012729025176
H	8.38634251368836	0.61024342536996	19.27116416248593
C	5.87285607181992	-1.01066660922019	17.36115052507289
H	5.17711288516263	-0.20083339302060	17.11162434636274
C	6.16700914244312	1.33948024371065	21.93183843628951
H	5.92866365573467	2.20975361289779	21.31410279691845
C	0.00535579172939	5.21912672673885	15.62040464395898
H	-0.48569548424878	4.99058331530168	14.68054743727411
C	1.25777256831534	5.82858110311113	18.06402950970881
C	-0.02475439910046	6.30363196108341	17.77249480894590
H	-0.53713308276366	6.92029299135063	18.50432992152095
C	1.91579202028628	6.21873045110197	19.37990954331190
H	2.85348052636054	5.66816412544440	19.49671510328075
C	-0.64827567811627	6.00020000406393	16.56706087685020
H	-1.64641474295218	6.37653008691762	16.36301871182198
C	7.53548044494303	1.59730132074165	22.58915445445741
H	7.81445596116554	0.76561679293421	23.24486312728457
H	7.49351111735186	2.50814590044673	23.19477313559077
H	8.31867997400924	1.71965669523456	21.83642639307815
C	1.03560666085407	5.84114485080456	20.58326452013931
H	1.55738122347364	6.08838651360252	21.51281968311575
H	0.82380379373893	4.76969066442639	20.58609752947674
H	0.08829943359800	6.39031068255871	20.57041683880934
C	1.97745881710893	3.85690355644557	14.80800418984385
H	3.05700784611076	3.84852774420267	14.99205004914477
C	5.05627866374668	1.23451077315439	22.99200968898782
H	4.08246374890664	1.09050184491056	22.51912454542160
H	5.01807432875652	2.15763728105009	23.57863295215004
H	5.24368640014848	0.40269030893901	23.67905389316696
C	11.71041359312226	2.06535412759029	17.84095303328302
H	11.20014563776776	1.64652712785915	16.96800032706388
H	12.70732284910770	1.61633730433893	17.90987749203155
H	11.83498302464642	3.14008813314194	17.67782784737398

C	10.91083641662699	1.77806938914867	19.13030460280963
C	5.25609596344899	-2.29752309425470	16.79625732408703
H	5.95231621971864	-3.14203364732018	16.84533871304313
H	5.00453036357944	-2.13987160625442	15.74355473322075
H	4.34155329208311	-2.57167214542743	17.33094730224079
C	1.48793673302663	2.40083572820279	14.92919684683526
H	0.41193521229043	2.34280536952456	14.72959116788247
H	1.67876292842767	2.00460146022188	15.93011189595456
H	2.01802568632464	1.76728658941119	14.21272480343987
C	1.78441611277424	4.38757050931729	13.37878388029280
H	2.38835943181082	3.79192601797932	12.68795464006762
H	2.09848830403680	5.43270098776715	13.29457212809610
H	0.74166581021693	4.31615239361338	13.05265952525705
C	2.25578002592512	7.72131019245291	19.38900857744749
H	1.34633234762267	8.32508562928462	19.29989935832490
H	2.92207563979423	7.98095920661390	18.56226771417095
H	2.75076807790816	7.98653809576415	20.32868833933248
C	10.81988018780883	0.25522888533916	19.31929576958867
H	10.28567141544522	-0.00763021223160	20.23865929584203
H	11.82908419609337	-0.16324807365370	19.38735413893550
H	10.31289316530884	-0.22438574912026	18.47533542555496
C	7.20283735809893	-0.69507410213461	16.64817965744000
H	7.60245378496441	0.27710129057776	16.94772147268904
H	7.04385472153928	-0.66292489672472	15.56675242206828
H	7.94949112049872	-1.46489596157698	16.87465923223920
C	6.08768002062515	9.66298962101694	15.09856191988178
H	6.37592195338410	10.10303763998822	16.05898955097865
H	5.85203471631505	10.48178526619543	14.41149830863585
H	6.94839131648994	9.12160571597866	14.69227701708293
C	11.65648724698905	2.38160208152350	20.34121286931230
H	11.76791989906555	3.46540335011563	20.23883915852882
H	12.65681580001327	1.94275384089518	20.42547348413780
H	11.11209130117413	2.18258364557833	21.26998908606675
C	4.49334376705791	8.19507792696517	13.84785664032376
H	5.30938156824649	7.58863014514928	13.44303863406480
H	4.29594755818485	9.02109328511443	13.15585806542902
H	3.59653808704314	7.56990102314136	13.89206991655035
C	3.68223767077634	9.58454077989063	15.78531935008454
H	2.77399930859002	8.98065892364230	15.87587479395107
H	3.46864662924999	10.41829552651769	15.10742142118713
H	3.91800451596651	9.99340997054244	16.77298863252092

1,9-Diamino-3,7-di-*tert*-butyl-N^1,N^9-bis(2,6-diisopropylphenyl)-10*H*-phenothiazin-10-yl radical **($SQH_2^{\bullet}$)**

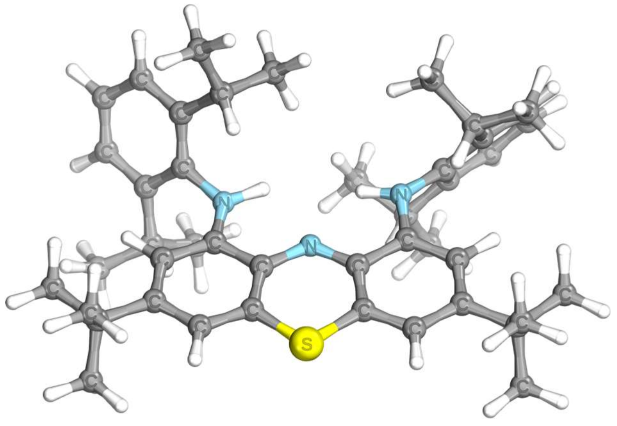

UTPSS-D3(BJ)/def2-TZVPP

xyz 0 2

C	-4.99003824990210	2.02905538092124	-3.20248026350544
C	-4.94971607144507	-3.50372897318766	-3.25452195378245
C	-2.66979884918198	2.91933971810323	-2.71906520868257
C	5.60767724673390	-3.38110394024140	-1.97934457606528
C	-3.76710157909059	1.92868848834624	-2.28211072773228
C	3.05598545999672	3.56327148424940	-2.26088565611666
C	-6.15588427494711	-2.11653122681196	-1.56212168335247
C	-4.97774290589162	-3.08156134171827	-1.76855327851298
C	-2.45757454531218	-3.19543698749616	-1.53208393784162
C	-3.63494567838840	-2.44476726681021	-1.40021014439549
C	-5.20900977608591	-4.33018206057810	-0.88869097796717
C	2.37116577028505	2.85490325705874	-1.08427378419536
C	-1.22290017475747	-2.65418661377712	-1.18293669467709
C	2.78641735220609	-3.18180383267815	-0.85455936599174
C	5.29182447824555	-3.03011288489992	-0.50840292169373
C	-4.09286640781785	2.14209981461497	-0.81149035365848
C	-3.54290708931462	-1.12972870899698	-0.93433700398526
C	1.49796165686963	-2.65531329815589	-0.80974276688921
C	-5.15806136217766	2.95208343920734	-0.40792726964173
C	1.06038513985906	3.57812859102665	-0.71418981557127
C	3.88687451179498	-2.42766757033975	-0.42172276241523
C	-1.10053030809340	-1.32606708443242	-0.69271243777678
C	6.37200294266217	-2.06409615556297	0.00342301276919
C	-2.31080716473902	-0.55755556684766	-0.60822313130842
C	5.34599126204492	-4.31685273469701	0.34522336497990
C	1.24162613392984	-1.34745415138808	-0.31695621549105
C	3.66285903462505	-1.14237696795677	0.08108347610088
C	3.26255907242379	2.71343700243046	0.13852625620442
C	2.37481488838598	-0.60779212001989	0.16419739076102
C	-3.27274384698085	1.58076246676880	0.18816619419813
C	4.22823962686423	3.67226744928575	0.45974028036804
C	-5.38659257849956	3.21986572798386	0.93997608974039
C	3.11160937048596	1.60596534994786	0.99677318560674
C	5.03850044015199	3.52057868010194	1.58112586037512
C	-3.49684644751812	1.82882602871834	1.55709093284063

C	-4.55721984285376	2.66840756870834	1.91210511383626
C	3.91229076416782	1.44297613178464	2.14534799648047
C	4.88406295129822	2.41196157837353	2.41049383518482
C	-2.58259145658982	1.21083520345000	2.60212067266867
C	-1.36220705259816	2.11925322969340	2.85405958014829
C	3.66007084979133	0.28560375858521	3.09995086844544
C	4.89969149165904	-0.12980860881092	3.90209575185597
C	-3.29339044544297	0.89457338066294	3.92501975432954
C	2.49036356791773	0.63540973265880	4.04117846108955
H	-4.70883278554182	1.74963047439686	-4.22281288095386
H	-4.79101685929187	-2.63376819676139	-3.89973299121684
H	-5.90192260617422	-3.97090010355166	-3.53037065778240
H	-2.38650617414531	2.74218838614334	-3.76243727667476
H	-5.38824393993337	3.04882609314489	-3.24101216699436
H	-4.14866109966986	-4.22221645982403	-3.45096754693760
H	-5.79407999068909	1.36259229401431	-2.87430090297305
H	2.40795054147392	3.52890154725778	-3.14253028508374
H	5.57899709985092	-2.48342039292898	-2.60499764756006
H	4.88660882702768	-4.09816697823676	-2.38234064461718
H	-3.02467839851730	3.95175415665677	-2.62795181537294
H	6.60725264805113	-3.82414572645569	-2.05393813292385
H	4.00663274066733	3.08404342250199	-2.51307819793279
H	-6.05743941994867	-1.22316247690415	-2.18724152862651
H	-3.35662440597758	0.92083218638966	-2.39159773850502
H	-7.08906293355677	-2.61858815150123	-1.83757323028897
H	-1.77980440816490	2.80353540337274	-2.09559319790872
H	3.25229639379368	4.61787958720944	-2.03985398933215
H	-2.49731159373211	-4.21562809382558	-1.90219667329946
H	-5.81022055969765	3.39043539491998	-1.15629182935131
H	-6.16242281263452	-4.80313761698212	-1.15011955344841
H	2.92900441309339	-4.18740054587692	-1.23898906029044
H	0.39020763591913	3.63253942620158	-1.57774955061986
H	2.11127646679935	1.84218910860544	-1.41369787292593
H	-4.41359434817056	-5.06833486798379	-1.02649372928046
H	-6.23973121602654	-1.79931270285917	-0.51767437471385
H	6.39467172777088	-1.13816090226611	-0.58025430302172
H	-4.43319446328576	-0.52660824279319	-0.80743764666606
H	4.61862394015048	-5.05688429224943	-0.00092674885721
H	7.35442094105685	-2.53992382122517	-0.08133756795210
H	6.34399306605618	-4.76571894929722	0.28555987827168
H	1.27128123443326	4.59762482265141	-0.37432823509424
H	4.36284249500617	4.53543354872453	-0.18450734346875
H	-5.23723493124144	-4.05589955970664	0.17059100732947
H	0.52959743729172	3.06701759318449	0.09589185430904
H	4.49094047734970	-0.52768564618273	0.41120150997355
H	1.17562398815172	0.95009404020258	0.47756322879005
H	6.21346420778171	-1.80514270769513	1.05529663559536
H	-6.21528073531305	3.85818963178334	1.23265445970645
H	5.12908515677010	-4.09294333731591	1.39457631528209
H	-1.26757336964780	0.92691043159054	0.21229663321048
H	5.79454210932346	4.26649198841652	1.80883447412793
H	-0.81075065372906	2.32311343682024	1.93018159753201
H	-4.74963684871245	2.87655185674909	2.95991110251614
H	-2.21396998977501	0.26422874655070	2.19024224369421
H	3.35041714932286	-0.57839902330429	2.50499549519555

H	5.75082560876917	-0.33684964326752	3.24551881794660
H	5.52009307800591	2.30678896829079	3.28336211662981
H	-1.68338264704995	3.08329957466605	3.26251541122098
H	-0.67268179338520	1.65415742650905	3.56543867309087
H	1.59389196709803	0.87529360961869	3.46416267078823
H	-4.18333833921180	0.28005320024021	3.75966154789549
H	4.68198165754717	-1.03595005594747	4.47631645595032
H	-3.59965567042653	1.80662877655457	4.44839167086095
H	5.20135365548985	0.64460299594226	4.61573542927061
H	2.74301019974024	1.50017765937605	4.66444064182096
H	-2.61564619990821	0.34832070484753	4.58887250071953
H	2.26188959858698	-0.20998742167347	4.69948577135479
N	0.03938212012726	-0.71873257168785	-0.27541681985006
N	-2.18127199426527	0.75153464548860	-0.19550212271038
N	2.11440850336973	0.63412021864447	0.70283127595943
S	0.18981619717682	-3.67493823663726	-1.38054030254116

UB3LYP-D3(BJ)/6-31G(d)

xyz 0 2

C	-5.02421098027635	2.01516828630564	-3.26844945270650
C	-4.97801692386029	-3.62340318553878	-3.17996644104625
C	-2.68727282651794	2.91440212520350	-2.87283463053966
C	5.57234197978529	-3.37508252529236	-2.03970902856531
C	-3.77363618637905	1.93864600409779	-2.38225979953503
C	3.04794944493856	3.59462291366028	-2.13051518868024
C	-6.17665608492585	-2.13510288477835	-1.57056194753221
C	-5.00584280531378	-3.12255462386409	-1.71863998789633
C	-2.48172423681109	-3.24517258975525	-1.48465429917222
C	-3.65515520969556	-2.48079693108620	-1.37721050745223
C	-5.25297836686441	-4.31694574051624	-0.76984306838828
C	2.33399600600777	2.85298124346071	-0.99151416033690
C	-1.24251747824492	-2.70261734570607	-1.15690595061999
C	2.79327703829158	-3.17965767740467	-0.86557074038537
C	5.30504140285241	-2.97633284526995	-0.57101690953213
C	-4.05637410422593	2.18476703591042	-0.90581621567741
C	-3.55272871806096	-1.14800782443085	-0.96201057909465
C	1.49590473895117	-2.68042924885363	-0.80106976492804
C	-5.08133577181851	3.04057555813676	-0.48925451768395
C	0.99197876464391	3.53850880958776	-0.66582930726702
C	3.88691467464772	-2.40369610151273	-0.44645723591928
C	-1.11623275489570	-1.36275148290960	-0.70426528094325
C	6.38493922791851	-1.96488819250971	-0.15013126981087
C	-2.31583315168174	-0.57384321414827	-0.65893345645293
C	5.42630177822068	-4.22519670271509	0.33103915801816
C	1.22763146905538	-1.37430489675710	-0.31268436585452
C	3.64365682231505	-1.13096027955003	0.08340267217478
C	3.18119576162940	2.71231513328873	0.26534633544275
C	2.34648742612031	-0.62074889698611	0.18351137376518
C	-3.23166554645027	1.60869971872173	0.08199486002158
C	4.10003777237235	3.69608361869628	0.64424856301143
C	-5.26723560838983	3.33376808671646	0.86060802925112
C	3.02647407263001	1.58428935721394	1.09961522515633

C	4.86535048563027	3.55239376362876	1.80018091808201
C	-3.41773470459056	1.87770648645496	1.45346705754363
C	-4.43708969612503	2.76257388328306	1.82189876574616
C	3.78694928905468	1.42692251814047	2.27558958881344
C	4.71190291515563	2.42480399506844	2.60254353491910
C	-2.49987512980594	1.24256269034265	2.48840093634541
C	-1.25343070810499	2.12005979470059	2.71778412956818
C	3.55769070710919	0.24933950904733	3.21206693207228
C	4.86589180435001	-0.40611703024679	3.67937324335287
C	-3.19680007885708	0.94473997702985	3.82372276973063
C	2.69951726920137	0.69456848187979	4.41085474614868
H	-4.77611756774297	1.70704797042966	-4.29036609518522
H	-4.79055702803723	-2.79355696813179	-3.87089604118146
H	-5.94001372586781	-4.07996525721215	-3.44388698329856
H	-2.44570812508916	2.72699876790159	-3.92623141104873
H	-5.42836447078355	3.03279241074792	-3.32626874903814
H	-4.19678119476393	-4.37377717202777	-3.33780797584956
H	-5.81868596045471	1.35681749843387	-2.89752791705983
H	2.44767288014158	3.53993214734792	-3.04563749759237
H	5.48412640810543	-2.50531423103672	-2.70046279051756
H	4.86862446222484	-4.13591062557592	-2.39162918813837
H	-3.02553160335529	3.95347676151629	-2.77750332193659
H	6.58542846502951	-3.78285752196064	-2.14244414194448
H	4.02995466812373	3.15566672275001	-2.33722705470282
H	-6.06982898099795	-1.27693843844074	-2.24407511440626
H	-3.36897149887776	0.92824867872508	-2.48406783214981
H	-7.11657854025114	-2.63996283494817	-1.82035364723387
H	-1.77143526520395	2.79660795212330	-2.28512718199225
H	3.19229185601426	4.65663528676974	-1.89963936389603
H	-2.53057636709382	-4.27592503778727	-1.82185433736712
H	-5.73411086933997	3.49594774703316	-1.22681782708203
H	-6.21394319016069	-4.79387261517383	-0.99916193666692
H	2.95144837154906	-4.18110643172299	-1.25359400879691
H	0.36576062901556	3.62213800934719	-1.56182146189937
H	2.11022315066596	1.84047756720755	-1.34798451768483
H	-4.46950145884558	-5.07601434082597	-0.86295817158798
H	-6.26387449902032	-1.75774372572634	-0.54559203644260
H	6.34523158524257	-1.05462204477664	-0.75876205162524
H	-4.43595323866190	-0.53198899630720	-0.86312674831330
H	4.70233184240419	-4.99728284034056	0.05011764465500
H	7.37682092444596	-2.41236091670137	-0.27995119168575
H	6.43088080207794	-4.65861599851458	0.25039982234771
H	1.15989508111282	4.54633918740364	-0.26790665206921
H	4.23218943471093	4.57686207920868	0.02385438304884
H	-5.27492848329709	-3.98472022366987	0.27420833770680
H	0.42341115072670	2.98304260013770	0.08858361908347
H	4.46057071065520	-0.50878040975972	0.42227941364247
H	1.10818557556392	0.89308825221224	0.58064537373965
H	6.28724284428473	-1.67718078878109	0.90247046130948
H	-6.06281398579328	4.00940928193695	1.16369580927880
H	5.24853984732730	-3.96440458032887	1.38054303376340
H	-1.24036178702447	0.94595516065578	0.04408055301317
H	5.58236816291498	4.32133218557521	2.07559374791262
H	-0.71382563880190	2.31300321852437	1.78367106212845
H	-4.59547687197294	2.99486596863847	2.87032942319725

H -2.15830178053264 0.28497301475305 2.07794679806826
H 2.98931646537870 -0.50787084236554 2.66861998711215
H 5.48183160193793 -0.71258985525943 2.82684863559922
H 5.30669788767766 2.32633933109501 3.50605072891398
H -1.53800940234381 3.09357815913679 3.13426179034131
H -0.55723569005653 1.63684958043231 3.41351714707898
H 1.74468736109226 1.11067043021856 4.07070687925840
H -4.10761441568329 0.35488490171986 3.67441201624652
H 4.64736330829225 -1.29751829999057 4.27875231286421
H -3.46995704213246 1.86265929323654 4.35720049948903
H 5.46503306721668 0.26859010965607 4.30191026733904
H 3.21296512292918 1.46620780324431 4.99734787713100
H -2.52460290944836 0.37739760903254 4.47731222959315
H 2.48802134385607 -0.15378035460677 5.07314351624431
N 0.02201789524116 -0.75577513216511 -0.28609136711471
N -2.16605127597447 0.75505658144471 -0.31980548222224
N 2.06066065995665 0.59907285252984 0.75663630701800
S 0.18094552270771 -3.73627740773232 -1.33321431253765

(*E*)-*N*-(3,7-di-*tert*-butyl-9-((2,6-diisopropylphenyl)amino)-10*H*-phenothiazin-1-ylidene)-2,6-diisopropylbenzenaminium bromide ([**QH$_2$**]**Br**)

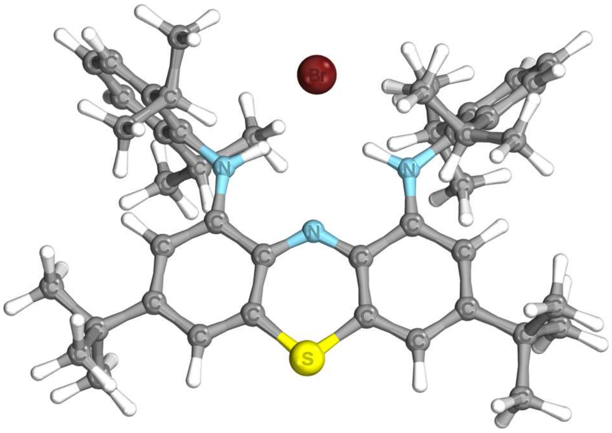

TPSS-D3(BJ)/def2-TZVPP

xyz 0 1

Br 5.16994136252114 9.33508786361812 5.79225976449426
S -1.32866379028446 9.38445893981666 7.98954380243871
N 1.61094153726933 9.12278919112650 6.99120001152592
N 3.78550746031775 8.96209457834507 8.62734860518335
H 3.90907715677128 9.06930699208220 7.59577476984994
N 2.35474835229196 9.06563580342259 4.36702308781106
H 3.09245399161066 9.12555154553681 5.10733166339924
C 0.06663587466783 9.23588142834254 3.66559835030566
H 0.40315583383352 9.20125173586930 2.63825844541707
C 1.39763455225988 9.09527207057286 8.30730809685225
C 0.65174069391497 9.21338716742282 6.07215162583680
C -0.75834405945676 9.32642789664382 6.34156405200805
C 1.05877608900513 9.17333738413866 4.67377083585662

C	2.56308199073043	8.95401458334922	9.17132865346020
C	3.72497103965031	8.75282659729319	0.41163886636575
H	4.07350434110710	8.67090222397935	-0.61396548193273
C	2.82803025423001	8.96255986215839	3.01778142910446
C	0.11111752858664	9.15147175674812	8.94449671077034
C	-1.68752906191495	9.39401741298124	5.32664258490504
H	-2.74154220826258	9.47559974843608	5.57167320649747
C	-1.27651548719559	9.34764693954914	3.96866294335455
C	2.36600737107790	8.80264298174950	10.56048318633423
H	3.24591023990859	8.65673576086269	11.17578294866823
C	2.77890415048403	7.70498905479991	2.38587822643503
C	4.98549259691335	8.71377703462210	9.36898774842277
C	3.23126124818731	7.62527955190384	1.06705914202095
H	3.20661415776473	6.67316415305909	0.54748477120286
C	5.51880483257745	9.72761890436083	10.18558109112450
C	3.78260384899849	9.97703123719376	1.06781950968915
H	4.17966864875781	10.84603799641792	0.55175770551135
C	-0.03100500645116	9.03096036282117	10.31372868280114
H	-1.02217324662556	9.06921499527429	10.74957605343516
C	3.33723663475552	10.10785749965380	2.38853025277066
C	5.60140137958746	7.46042723421289	9.21609169315144
C	-2.34444674155607	9.42388833701089	2.87781784997098
C	-3.11528044907484	10.75616081563886	3.02092018716363
H	-3.59997698906692	10.83938065526093	3.99802366984889
H	-3.89132112256491	10.81818589645832	2.25068728945086
H	-2.43985310250000	11.60877781686481	2.90111431213797
C	6.68149914246099	9.43438140456255	10.90379763592521
H	7.11967979950172	10.19159729297345	11.54581369593474
C	1.10503908527648	8.83812228431263	11.13431676117197
C	3.37284900278796	11.45090997201732	3.09656225948460
H	3.18922937745343	11.26857910154418	4.15882062063755
C	2.31048574144233	6.48117508378445	3.15737882793406
H	1.48762668034383	6.79609611855448	3.80882309095101
C	7.29709650802465	8.18948284216603	10.79110059746888
H	8.20250982470228	7.98248300606162	11.35451142876481
C	4.89614754330882	11.11371888210842	10.19767697527273
H	3.81873864828577	10.99763528408636	10.04272634048426
C	-1.74153772948647	9.35563464448478	1.46615817695991
H	-1.05613826851294	10.18815907558255	1.27885321909278
H	-2.54691974865128	9.41035878628896	0.72715167464265
H	-1.19915674250068	8.41868648404110	1.30439379576930
C	6.76813000693693	7.21726931559414	9.94748218842249
H	7.26833306809426	6.25865763531347	9.85294052119571
C	-3.32614464912331	8.24230554371997	3.05043392327610
H	-2.80197195584162	7.28647308991728	2.95381570076605
H	-4.10150282635743	8.29146001457433	2.27858061663649
H	-3.81853874450981	8.26437104974911	4.02694662619621
C	4.75119062446937	12.11975701115583	2.98950442322569
H	5.52018078302254	11.46760416923061	3.41059048421792
H	4.75158543993627	13.05996197044139	3.55101308178279
H	5.00929793645951	12.35167623571891	1.95019577030829
C	4.98516447895705	6.40126822344208	8.31711059985663
H	4.36482516514485	6.92091960652452	7.58142772291679
C	3.45024802003398	5.96872506937254	4.06299031365103
H	4.28147504538395	5.60271365620563	3.45076279189505

H	3.09518502899541	5.14336317514107	4.69073033895791
H	3.83765508926972	6.76225372713696	4.70825228060516
C	2.25553369127933	12.36895260700189	2.56429922244636
H	2.39626556932619	12.57443841550174	1.49704965550940
H	2.26019108311353	13.32512171091781	3.09870592538902
H	1.27034907127432	11.90850112119135	2.69287383301367
C	5.45087833225395	11.93403980766114	9.01414517411735
H	6.51751964096188	12.13448242448579	9.16490229349730
H	4.92850038117877	12.89451284850320	8.94027483705835
H	5.34301184748401	11.39727412331395	8.06699745827062
C	5.09080635531066	11.86258640922502	11.52228423934116
H	4.73727141440926	11.27446318745345	12.37532998291410
H	4.53274137838579	12.80416890897420	11.50084265324376
H	6.14331385166870	12.11195856231448	11.69344366819245
C	6.03386193272644	5.60752328131907	7.52789148644054
H	6.66314974179804	6.28319560972715	6.94299698855746
H	5.53298339293821	4.92158120179208	6.83672436597469
H	6.67313292880825	5.00652987543624	8.18413748433498
C	1.77541198566598	5.35852035467316	2.26043575112055
H	0.98640565209982	5.71874393537855	1.59183538590859
H	1.36107013980747	4.55636817568036	2.87954239652091
H	2.56888607751573	4.91909877012755	1.64657239812748
C	4.07225175993324	5.47048181870571	9.13919926586402
H	4.65272357020438	4.92640015024445	9.89285774897754
H	3.58595685853501	4.73731901792805	8.48624208876727
H	3.29333723123810	6.03792576428549	9.65878287276868
C	0.97474310916476	8.65553463305816	12.64944496151874
C	-0.48068582382394	8.75090596507159	13.13355369030099
H	-0.50862508584367	8.61736895804996	14.21935552156076
H	-1.10844366971356	7.97346997994397	12.68583850411174
H	-0.91954370768225	9.72798045278095	12.90572161259536
C	1.53070862264836	7.26419124042342	13.03050231555121
H	2.58079460269904	7.16227300529204	12.74341066298447
H	0.96306357071656	6.47041036123887	12.53479015997408
H	1.45539170934539	7.11911141504256	14.11357494473324
C	1.80221352643807	9.74793880597354	13.36367561976561
H	2.86048968623124	9.68436385256326	13.09814137136914
H	1.71472791944213	9.62765592265812	14.44876692222461
H	1.44285537432549	10.74643956065669	13.09579770890523

3 References

[1] A. Kormos, I. Moczar, A. Sveiczer, P. Baranyai, L. Parkanyi, K. Toth, P. Huszthy, *Tetrahedron* **2012**, *68*, 7063-7069.

[2] K. Yamamoto, S. Higashibayashi, *Chem-Eur J* **2016**, *22*, 663-671.

[3] J. Mosnáček, R. Nicolaÿ, K. K. Kar, S. O. Fruchey, M. D. Cloeter, R. S. Harner, K. Matyjaszewski, *Industrial & Engineering Chemistry Research* **2012**, *51*, 3910-3915.

[4] Y.-R. Luo, *Comprehensive handbook of chemical bond energies*, Taylor & Francis, Boca Raton, Fla., **2007**.

[5] G. R. Fulmer, A. J. M. Miller, N. H. Sherden, H. E. Gottlieb, A. Nudelman, B. M. Stoltz, J. E. Bercaw, K. I. Goldberg, *Organometallics* **2010**, *29*, 2176-2179.

[6] Bruker TopSpin 4.0.7, https://www.bruker.com/products/mr/nmr/software/topspin.html.

[7] D. F. Evans, *J. Chem. Soc.* **1959**, 2003-2005.

[8] a) Z. Otwinowski, W. Minor, *Method. Enzymol.* **1997**, *276*, 307-326; b) G. H. L. Zsolnai, *XPMA, Heidelberg, Ruprecht-Karls-Universität* **1994**.

[9] B. C. APEX3, **2019**.

[10] a) G. M. Sheldrick, *Acta Crystallogr. A* **2015**, *71*, 3-8; b) B. C. N. M. Ruf, *Application Note SC-XRD 503, Bruker AXS GmbH, Karlsruhe, Germany* **2014**; c) G. M. Sheldrick, *SHELXL-2018, Program for Structure Refinement, Georg-August-Universität, Göttingen* **2014-2018**.

[11] O. V. Dolomanov, L. J. Bourhis, R. J. Gildea, J. A. K. Howard, H. Puschmann, *J. Appl. Crystallogr.* **2009**, *42*, 339-341.

[12] G. M. Sheldrick, *Acta Crystallogr. A* **2008**, *64*, 112-122.

[13] F. Neese, *Wires. Comput. Mol. Sci.* **2012**, *2*, 73-78.

[14] F. Neese, F. Wennmohs, A. Hansen, U. Becker, *Chem. Phys.* **2009**, *356*, 98-109.

[15] a) F. Neese, *J. Comput. Chem.* **2003**, *24*, 1740-1747; b) K. Eichkorn, O. Treutler, H. Ohm, M. Haser, R. Ahlrichs, *Chem. Phys. Lett.* **1995**, *242*, 652-660.

[16] S. Grimme, J. Antony, S. Ehrlich, H. Krieg, *J. Chem. Phys.* **2010**, *132*.

[17] a) S. Grimme, S. Ehrlich, L. Goerigk, *J. Comput. Chem.* **2011**, *32*, 1456-1465; b) E. R. Johnson, A. D. Becke, *J. Chem. Phys.* **2005**, *123*; c) A. D. Becke, E. R. Johnson, *J. Chem. Phys.* **2005**, *123*.

[18] J. M. Tao, J. P. Perdew, V. N. Staroverov, G. E. Scuseria, *Phys. Rev. Lett.* **2003**, *91*.

[19] a) F. Weigend, R. Ahlrichs, *PCCP* **2005**, *7*, 3297-3305; b) F. Weigend, *Phys. Chem. Chem. Phys.* **2006**, *8*, 1057-1065.

[20] a) A. D. Becke, *Phys. Rev. A* **1988**, *38*, 3098-3100; b) A. D. Becke, *J. Chem. Phys.* **1993**, *98*, 1372-1377.

[21] a) W. J. Hehre, R. Ditchfield, J. A. Pople, *J. Chem. Phys.* **1972**, *56*, 2257-+; b) P. C. Hariharan, J. A. Pople, *Theor. Chim. Acta* **1973**, *28*, 213-222; c) J. S. Binkley, J. A. Pople, W. J. Hehre, *J. Am. Chem. Soc.* **1980**, *102*, 939-947; d) R. Krishnan, J. S. Binkley, R. Seeger, J. A. Pople, *J. Chem. Phys.* **1980**, *72*, 650-654; e) M. M. Francl, W. J. Pietro, W. J. Hehre, J. S. Binkley, M. S. Gordon, D. J. Defrees, J. A. Pople, *J. Chem. Phys.* **1982**, *77*, 3654-3665; f) M. J. Frisch, J. A. Pople, J. S. Binkley, *J. Chem. Phys.* **1984**, *80*, 3265-3269.

[22] J. Hioe, D. Šakić, V. Vrček, H. Zipse, *Org. Biomol. Chem.* **2015**, *13*, 157-169.

[23] G. Knizia, *J. Chem. Theo. Comp.* **2013**, *9*, 4834-4843.
